# Alzheimer’s disease rewires gene coexpression networks coupling different brain regions

**DOI:** 10.1038/s41540-024-00376-y

**Published:** 2024-05-09

**Authors:** Sanga Mitra, Kailash BP, Srivatsan C R, Naga Venkata Saikumar, Philge Philip, Manikandan Narayanan

**Affiliations:** 1 Bioinformatics and Integrative Data Science group, Department of Computer Science and Engineering, Indian Institute of Technology (IIT) Madras, Chennai, India; 2 Centre for Integrative Biology and Systems Medicine, IIT Madras, Chennai, India; 3 Robert Bosch Centre for Data Science and Artificial Intelligence, IIT Madras, Chennai, India; 4 Sudha Gopalakrishnan Brain Centre, IIT Madras, Chennai, India

**Keywords:** Regulatory networks, Neuroscience, Systems analysis, Computational biology and bioinformatics

## Abstract

Connectome studies have shown how Alzheimer’s disease (AD) disrupts functional and structural connectivity among brain regions. But the molecular basis of such disruptions is less studied, with most genomic/transcriptomic studies performing within-brain-region analyses. To inspect how AD rewires the correlation structure among genes in different brain regions, we performed an Inter-brain-region Differential Correlation (Inter-DC) analysis of RNA-seq data from Mount Sinai Brain Bank on four brain regions (frontal pole, superior temporal gyrus, parahippocampal gyrus and inferior frontal gyrus, comprising 264 AD and 372 control human post*-*mortem samples). An Inter-DC network was assembled from all pairs of genes across two brain regions that gained (or lost) correlation strength in the AD group relative to controls at FDR 1%. The differentially correlated (DC) genes in this network complemented known differentially expressed genes in AD, and likely reflects cell-intrinsic changes since we adjusted for cell compositional effects. Each brain region used a distinctive set of DC genes when coupling with other regions, with parahippocampal gyrus showing the most rewiring, consistent with its known vulnerability to AD. The Inter-DC network revealed master dysregulation hubs in AD (at genes *ZKSCAN1*, *SLC5A3*, *RCC1, IL17RB, PLK4*, etc.), inter-region gene modules enriched for known AD pathways (synaptic signaling, endocytosis, etc.), and candidate signaling molecules that could mediate region-region communication. The Inter-DC network generated in this study is a valuable resource of gene pairs, pathways and signaling molecules whose inter-brain-region functional coupling is disrupted in AD, thereby offering a new perspective of AD etiology.

## Introduction

The human brain connectome is comprised of a functional and a structural connectome, which are essentially large-scale networks linking distinct brain regions, mapped using different neuroimaging techniques^1,2^ (e.g., functional Magnetic Resonance Imaging or fMRI maps functional connectivity from correlations of brain activity measurements, and diffusion MRI or dMRI maps structural or anatomical connectivity from white matter tract measurements). The structural connectome can influence the functional connectome shaping brain region specific activity^3^; and includes two major types of intercellular communication found in the central nervous system: wired transmission (synaptic point-to-point communication between neurons) and volume transmission (extra-synaptic transmission between neurons or neurons and glia through cerebrospinal fluid and extracellular fluid)^4,5^. Normal brain activity relies on the overall connectome map, which disease can rewire and disrupt i.e., alter the functional connectivity between brain regions^6^.

Investigating the brain connectome revealed the abnormalities in brain connectivity of progressive neurodegenerative diseases such as Alzheimer’s disease (AD)^7,8^, which is characterized by the extracellular amyloid beta (Aβ) plaque development and intracellular neurofibrillary tangles (NFTs) formation at the molecular level, and manifests as memory loss, cognitive dysfunction, and social disorders at the clinical level^9^. Genome-wide association studies (GWAS) of AD have identified risk loci and potential causative genes^10,11^, but which brain regions and mechanisms these genes act through is not fully characterized. Lack of understanding about molecular changes in the brain connectome hampers therapeutic interventions aimed at slowing down or halting neuronal loss associated with AD.

Genomic studies are becoming instrumental to understand the molecular basis of neural circuits^12,13^ connecting different brain regions in health and disease. Brain functional connectivity is known to be under genetic control^14,15^, and recent studies are linking gene expression to connectome data^16,17^. Transcriptomic analysis have elucidated gene regulatory interactions operating within brain tissues or regions of healthy/diseased individuals. For instance, the effect of AD on different cortical regions has been studied using gene-gene coexpression (correlation) network and module-trait network analyses^18^. Furthermore, many established differential expression (DE) studies have identified individual genes whose expression is affected by disease in a region-specific manner. Brain region-specific coexpression network analysis combined with GWAS studies has also revealed significant AD genes^19^. Gene coexpression modules prevalant in all brain regions or specific to one region in different neuropsychiatric disease have also been explored^20,21^. As of now most AD gene expression studies^22,23^, have mainly focused on within-tissue/within-region analysis to provide insights into disease genes/processes. Therefore, the molecular mechanisms supporting inter-brain-region connectivity, i.e., gene-expression coordination across brain regions, especially in neurodegenerative disease states, remain undefined. How genes from one brain region can affect another brain region, how the inter-brain-region communication occurs needs to be explored. While tissue-tissue communication has been studied before^24,25^, brain region communication on gene level is rarely explored.

To understand the gene-gene couplings across brain regions under normal vs. disease conditions, we constructed a differential correlation (DC) network across four brain regions using multi-region transcriptomic (specifically RNA sequencing, RNA-seq) data from the Mount Sinai Brain Bank (MSBB) based study^26^. We account for cellular composition effects in the data to better capture cell-intrinsic changes in disease. The Inter-DC network is comprised of numerous gene pairs whose correlation strength is altered (lost or gained) in disease (AD) group compared to control (CTL) group. Interestingly, each brain region uses a unique set of genes when interacting with genes in other brain regions. The rewired Inter-DC gene pairs is most prominent for coupling of parahippocampal gyrus with other brain regions, in accordance with earlier studies on vulnerability^27^ or white matter degeneration^28^ of different brain regions.

Bipartite network clustering and associated analysis of the overall network of Inter-DC relations uncovered dysregulated gene-gene interactions, and identified biological processes related to synaptic signaling, regulation of synaptic vesicle cycle and neurogenesis as the most affected processes across brain regions. Systematically screening the Inter-DC network for hub genes revealed an AD-GWAS signal enriched gene *ZKSCAN1*^29^ as a dominant (dys)regulator, with its DC partner genes clarifying *ZKSCAN1*’s hypothesized role and mechanism in AD pathogenesis. Some of our Inter-DC modules are enriched for different types of signaling molecules, such as ligand-receptor molecules, AD-CSF markers (Cerebro-Spinal Fluid), secreted proteins, and neurotransmitters-neuroreceptors. These suggest plausible mechanistic hypotheses of information transfer across brain regions that are affected by AD. Taken together, our results from assessing the effect of AD on the network of inter-brain-region gene-gene correlations furnish us with vital molecular information about AD pathogenesis that may help in promoting AD therapeutics.

## Results

### Gene pairs between brain regions are rewired in AD pathology

**Fig. 1:**
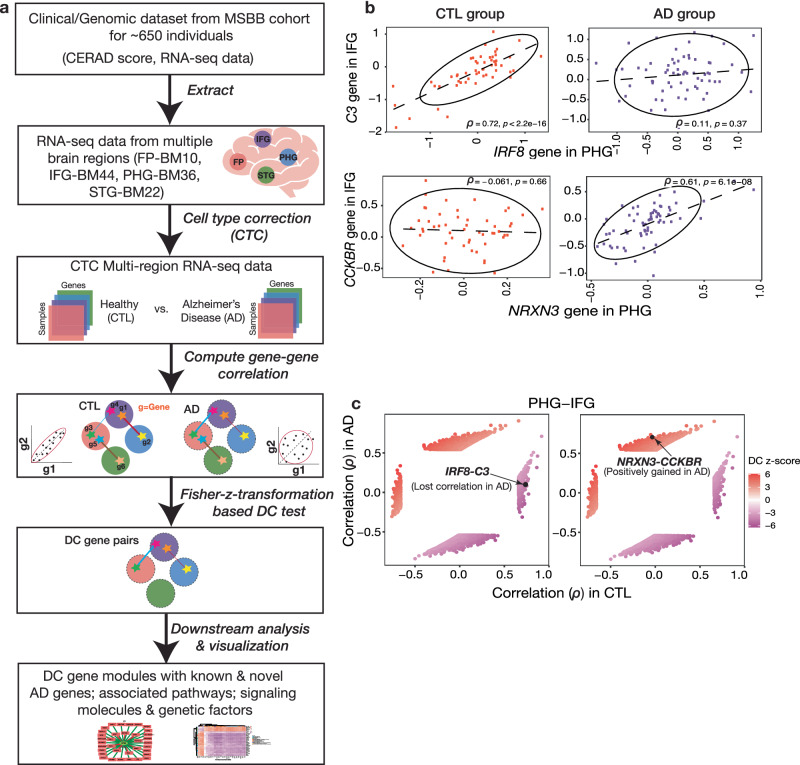
Gene pairs are differentially correlated (DC) in inter-brain-region comparison in AD pathology. **a** Schematic of our methodology—To understand inter-brain-region dysregulation, we obtained RNA-seq gene expression data from MSBB for four different brain regions (details in text); grouped them into AD and CTL samples based on CERAD (Consortium to Establish a Registry for AD) score, and computed Spearman’s correlation between all pairs of genes in each pair of brain regions separately in the AD group and the CTL group. By correcting the expression data for cell-type composition effects before Inter-DC analysis, the confounding influence of cell-type proportions causing DC patterns is mitigated (we specifically used a CellCODE model for this cellular deconvolution based on 80 marker genes (MG); i.e., 20 MG each for the four major cell types, was identified as the best performing model and hence used to estimate the relative frequencies of the cell types; details in “Methods”). **b** Gene pairs gained (*IRF8*-*C3* gene pair) or lost (*NRXN3*-*CCKBR*) correlation in the AD group relative to CTL samples. All such rewired gene pairs constitute the altered gene network in AD pathogenesis. The ellipses in these plots are generated using R ggplot2 stat_ellipse() function using default arguments (95% confidence interval based on a multivariate t-distribution fit to the data). **c** The Inter-DC pairs form four distinct clusters in a scatter plot representing the different categories of changes detected from the AD vs. CTL Inter-DC analysis. Comparing the size of the two gained correlation clusters (both positively and negatively gained) with the lost correlation clusters, there are more gene pairs that gained correlation (see Methods and Supplementary Table 2).

Studying how a network of gene-gene correlations (coexpression patterns) observed in a group of healthy individuals gets altered in a disease group is a good starting point to understand the molecular disruptions caused by the disease condition^30^. Inter-DC analysis identifies such gene pairs that have either gained or lost correlation strength significantly in the disease compared to control group. Performing Inter-DC analysis (see Fig. 1a and Methods) on RNA-seq data from MSBB^26^ of 372 CTL vs. 264 AD samples of four brain regions; namely Brodmann Area (BM10) - frontal pole (FP), BM22 - superior temporal gyrus (STG), BM36 - parahippocampal gyrus (PHG), and BM44 - inferior frontal gyrus (IFG); we observed significant rewiring of gene pairs between two brain regions in AD compared to CTL samples (Table 1, Fig. 1b). Details of all DC gene pairs discovered at FDR 1% are in Supplementary File 1 (note that the terms *DC* and *Inter-DC* are used interchangeably in this work).

**Table 1:** For each inter-brain-region comparison, the number of DC gene pairs (edges) and unique DC genes (nodes) in the DC network detected at FDR 1% are reported (A DC gene is any gene participating in at least one DC relation; BR1 and BR2 stands for Brain Region 1 and 2 respectively; and DC Dysregulation index is the percentage of detected DC pairs out of all tested gene pairs)

Brain Region Pair (BR1-BR2)	Total DC edges	BR1- # DC Genes	BR2- # DC Genes	DC Dysregulation percentage (%)	DC edges driven by DE
FP-STG	2961	2013	1844	2.54%	266 (9%)
FP-PHG	2629	1597	1409	5.41%	169 (6%)
FP-IFG	9962	4609	4235	3.40%	741 (7%)
STG-PHG	6274	2580	2549	8.94%	218 (3%)
STG-IFG	8179	3297	3548	5.24%	173 (2%)
PHG-IFG	12979	3642	4202	14.01%	141 (1%)

A DC edge is said to be driven by DE if any of the two genes in this DC edge or both are DE.

Interestingly, our inter-brain-region comparison of PHG-IFG, the two most vulnerable of these four regions according to an earlier study^27^, shows the maximum rewiring of gene pairs (12,979) compared to the other five inter-brain-region comparisons (Table 1). To clarify this further, we also calculated the percentage of detected DC pairs (at FDR 1%) out of all tested gene pairs. This measure, which we call DC Dysregulation percentage, attained a maximum of 14.01% for the same PHG-IFG region pair, and a minimum of 2.54% for FP-STG (Table 1). This indicates that gene interactions between the two most vulnerable brain regions in AD are most affected, whereas those between less vulnerable regions (FP-STG) are the least affected. Notably, the DC dysregulation percentage also declined depending on the decreasing vulnerability rank of the brain regions interacting with PHG. Note that PHG was reported as the most vulnerable site in AD^27^, and also exhibited prominent white matter tract degeneration^28^.

For each significant DC gene pair, it has either lost or gained correlation in AD compared to CTL (e.g., Fig. 1b). Further, delineating a DC pair based on a z-score threshold yielded 4 categories (Fig. 1c, Supplementary Fig. 1a, and Supplementary Table 1), which can be grouped into 3 classes: gained positive correlation (PG), lost correlation (LC), and gained negative correlation (NG). The distribution of DC edges in these 3 categories are noted in Supplementary Table 2. DC edges with gained correlation outnumber those with lost correlation. The position and class of DC for the gene pairs, *IRF8-C3* and *NRXN3-CCKBR* from PHG and IFG, are highlighted in Fig. 1c. NRXN3^31^ and C3^32^ proteins in CSF are already reported as biomarkers for AD, suggesting that CSF (along with ISF (Interstitial Fluid)) may act as a medium enabling the normal coupling of certain gene pairs between two brain regions and its rewiring in AD. This further indicates that volume transmission is required to maintain inter-brain-region gene-gene network. Furthermore, assessing overlap of DC gene sets from all six inter-brain-region comparisons resulted in only a few gene pairs that are common across these comparisons (Supplementary Fig. 1b). This indicates that the rewiring of gene pairs varies based on which pair of brain regions are considered for DC analysis.

### DE genes do not drive DC gene pairing

Since DE-based vulnerability index from an earlier study and our DC-based dysregulation index provide similar rankings for how the different brain regions are affected by AD, we wanted to check if DC results are driven by DE or if they complement DE. We checked the overlap between DC and DE genes for every inter-brain-region comparison. In this study, we have used cell type corrected (CTC) DEGs for comparison since CTC data is also used for computing DC. Significantly altered CTC-DEGs are compared with DC genes participating in all six inter-brain-region comparisons at FDR 0.05, 0.1, and 0.2. Even at a relaxed cut-off of FDR 0.2, more than 90% of DC gene pairs are not driven by DEG **(**Table 1**)**. While testing how many DC edges overlap with DEGs, it became evident that 9% of edges are driven by DEG for FP-STG comparison, whereas for PHG-IFG, only 1% are affected. This shows that DEGs do not confound DC relations.

Additionally, to substantiate our argument, we conducted a comparison between the aggregate DE score and DC z-score for the tested gene pairs in a given inter-brain-region comparison. Supplementary Fig. 1a vividly demonstrates that as the DC score increases, the DE score decreases (as depicted by the orange dots), underscoring the complementary nature of DC to DE. Furthermore, we offer a detailed elucidation of this relationship using two gene pairs for clarity (Supplementary Figs. 1b, 2c).

### Inter-DC network contains region-exclusive interactions, and hubs of AD dysregulation

**Fig. 2:**
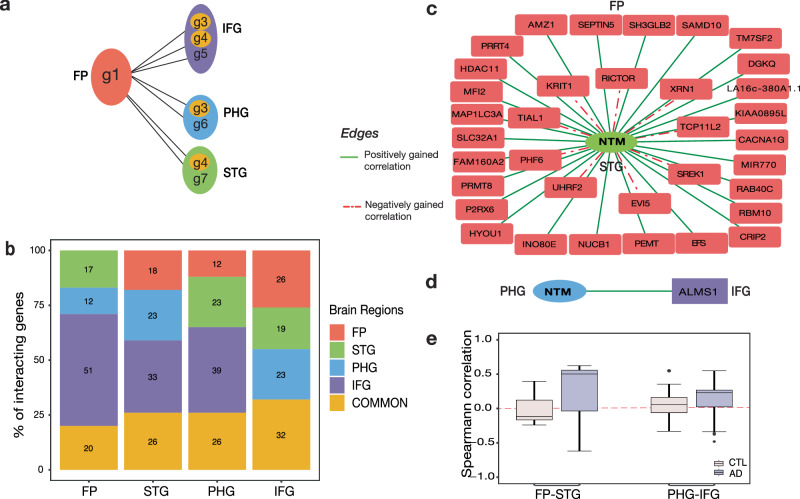
In the Inter-DC network, each brain region uses a distinct gene profile to interact with other brain regions. **a** Schematic Inter-DC network between Frontal Pole (FP) and other three brain regions, depicting distinctive dysregulation patterns. **b** Stacked bar graph denotes that each brain region has an exclusive set of genes mostly when interacting with other brain regions in the DC network. **c** Hub gene *NTM* from STG is differentially correlated with 35 genes in FP. The edge color represents the category of Inter-DC relation. Green (solid line) represents positively gained, and red (dashed line) represents negatively gained. **d** In this network, *NTM* from PHG is differentially correlated (positively gained in DC) to only one gene *ALMS1* in IFG. **e** The Spearman correlation coefficients between the hub gene *NTM* and its Inter-DC partner genes (noted in panel **c**, **d**) are shown for the CTL and AD conditions as boxplots, both for FP-STG and PHG-IFG inter-brain-region comparisons. Boxplots in this figure and elsewhere in this paper show the median as the center line, upper and lower quartiles as box limits, 1.5x interquartile range as whiskers, and outliers outside the whiskers as points.

For each inter-brain-region BR1-BR2 (BR1 stands for brain region 1 and BR2 for brain region 2) comparison, we sought to identify whether the DC genes were exclusive to or shared among the two regions. We detected only 7–21% gene overlap between the BR1 vs. BR2 DC genes (Supplementary Fig. 3). Inspecting whether these common genes in each inter-brain-region analysis had the same gene neighbors in both brain regions, we found it not to be the case surprisingly (Supplementary Table 3). Next, we pooled the genes that a given brain region (say FP) uses to interact with the three other brain regions (STG, PHG, and IFG) in the DC networks (Fig. 2a), and realized that only 20–32% of these DC genes are common across at least two regions—the remaining genes constituting a large fraction of all pooled DC genes are exclusive to a region (Fig. 2b). DC edges shared by one brain region with the other three brain regions are illustrated in Supplementary Fig. 4, showcasing both the overlapping and unique DC edges across inter-brain region comparisons. It seems a complex interplay between genes and region-specificity influences the activity of genes and their involvement in disease pathology. Further, this region-exclusive interaction can also be attributed to volume transmission, because diffusion of neuroactive substance across extracellular space, responsible for inter-brain-region communication, is often heterogenous and anisotropic^5^. Together these analyses reinforce the importance of focusing on multiple brain regions and associated gene-gene interactions to understand AD etiology.

To further understand the inter-brain-region interactions dysregulated in AD, we focused on the hub genes which participate in a large number of DC relations, and tested if they are shared or exclusive across inter-brain-region analyses. We are interested in gene hubs as they can underpin structural connectome hubs, due to the link between gene expression and neuronal activity^12^. When examining the degree (number of DC interaction partners) of each gene for each inter-brain-region comparison (Supplementary Fig. 5), we observed only a few hub genes, with more than 50% being non-hub having unit degree (single DC interaction) out of 15,905 DC genes. Moreover, the highest degree of hub gene in each brain region ranges from 20 (FP in FP-STG) to 113 (IFG in FP-IFG) (Supplementary Table 4). However, we noticed that a few hub genes with the highest degree are lncRNAs, pseudogenes, and antisense RNAs. Since their functionality is not well documented, we decided to select the protein-coding genes for our hub-gene analysis (Table 2). Interestingly, gene *ZKSCAN1* (Zinc Finger With KRAB And SCAN Domains 1) is found to act as a hub gene (309 unique DC partners) for different inter-brain-region comparisons. Top 20 hub genes selected from all 6 inter-brain-region DC interactions based on number of DC partners and in how many of six inter-brain-region comparisons they are present at FDR 1% are noted in Supplementary File 1. *ZKSCAN1* is reported to have a role as a transcription factor that modulates GABA type-A receptor expression in the brain^33^. Exploring DC partners of *ZKSCAN1*, we realized it has region-exclusive partners mostly. Two of its DC partner genes, *SGK2* (Serum/Glucocorticoid Regulated Kinase 2) and *TCF12* (Transcription Factor 12), are found in four inter-brain region comparisons (FP-IFG, STG-PHG, STG-IFG and PHG-IFG), where the DC edge *ZKSCAN1-SGK2* is positively gained in all four and *ZKSCAN1-TCF12* is negatively gained in all four analyses. While *SGK2* is known to regulate ion channel transport and transport of glucose, metal ions^34^, etc., its involvement in AD is not known. On the other hand, *TCF12*, required for the initiation of neuronal differentiation^35^, is known to be dysregulated in AD^36^. *PPDPF* (Pancreatic Progenitor Cell Differentiation And Proliferation Factor) that acts as a hub gene in STG for STG-PHG interactions is also a DC partner of *ZKSCAN1*. Though, not the highest connected hub gene in any of the six inter-brain-region comparisons, genes *SLC5A3* (Solute Carrier Family 5 Member 3), *TFCP2* (Transcription Factor CP2) and *RCC1*(Regulator Of Chromosome Condensation 1) have second highest (total 187), third highest (total 159) and fourth highest (total 156) DC gene partners after *ZKSCAN1*. All these genes act as DC partners of *ZKSCAN1*. *TFCP2* is only connected in PHG-IFG and *RCC1* in STG-PHG whereas *SLC5A3* is connected in 3 inter-brain-region interactions. This shows the top hub genes are highly connected among themselves, dictating the change in inter-brain-region DC gene network in AD pathology.

**Table 2:** Hub protein-coding genes along with their degree in each inter-brain-region comparison (see also Suppl. Table 4)

Brain Region Pair	BR1			BR2		
	Highest Degree	Gene Symbol	Gene Name	Highest Degree	Gene Symbol	Gene Name
FP-STG	17	*TBC1D30*	TBC1 Domain Family Member 30	35	*NTM*	Neurotrimin
FP-PHG	27	*BFAR*	Bifunctional Apoptosis Regulator	42	*LZTS1*	Leucine Zipper Tumor Suppressor 1
FP-IFG	54	*LDB3*	LIM Domain Binding 3	76	*ZKSCAN1*	Zinc Finger With KRAB And SCAN Domains
STG-PHG	42	*PPDPF*	Pancreatic Progenitor Cell Differentiation And Proliferation Factor	55	*ZKSCAN1*	Zinc Finger With KRAB And SCAN Domains
STG-IFG	48	*FSD1*	Fibronectin Type III And SPRY Domain Containing 1	86	*PLK3*	Polo Like Kinase 3
PHG-IFG	111	*IL17RB*	Interleukin 17 Receptor B	82	*ZKSCAN1*	Zinc Finger With KRAB And SCAN Domains

Moreover, *NTM* (Neurotrimin), *LZTS1* (Leucine Zipper Tumor Suppressor 1), and *FSD1* (Fibronectin Type III And SPRY Domain Containing 1) are among a few other hub genes detected. The different DC partners of the hub gene *NTM* from FP-STG comparison is shown in Fig. 2c; interestingly, in PHG-IFG comparison, *NTM* in PHG is DC with only one gene, *ALMS1* (*ALMS1* Centrosome And Basal Body Associated Protein) in IFG (Fig. 2d). This observation highlights that even the same gene from different regions have distinctive patterns of disease dysregulation. To verify that the region exclusivity is not biased by stringent FDR cut off and is due to actual molecular changes imparted by disease, we assembled gene-gene correlations (*NTM* vs. all its DC partner genes denoted in Fig. 2c, d) for both inter-brain-region comparisons (FP-STG and PHG-IFG) in CTL and AD conditions and represented as box plot in Fig. 2e. It is clearly evident from Fig. 2e that NTM-DC partner gene correlation is significantly different between CTL and AD in FP-STG compared to PHG-IFG. This justifies the DC behavior of *NTM* gene in different inter-brain-region comparisons. Such dominant region-exclusive DC relations is due to the disease affecting different regions in different ways, as well as the coexpression networks in healthy control states being region-specific to begin with (i.e., same genes in multiple brain regions having different coexpression relations with genes from another region, possibly due to the different spatial/molecular context they are in).

### Bipartite network clustering reveals pathways with disrupted inter-brain-region connectivity

**Fig. 3:**
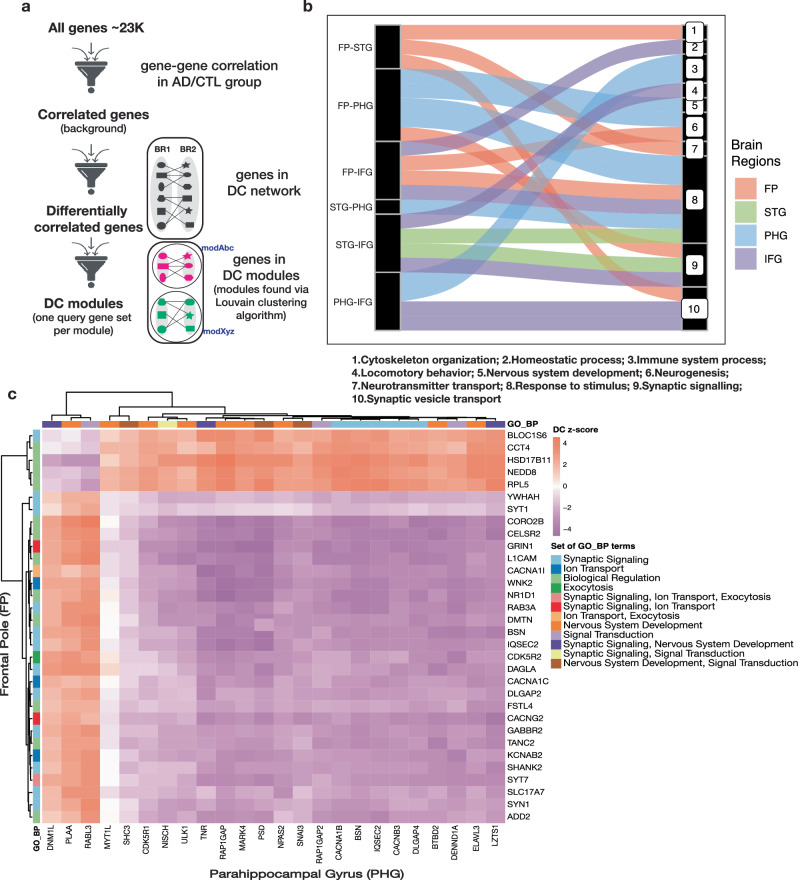
Bipartite Inter-DC modules provide insights into the inter-brain-region biological processes affected by AD. **a** The union of correlated gene pairs in AD and that in CTL are tested for DC, and the resulting bipartite (two-region) Inter-DC network partitioned into modules using the Louvain method (see “Methods”). To identify biological pathways and potential signaling factors enriched in each of these Inter-DC modules, we perform Over Representation Analysis (ORA; see “Methods”) with background gene set being all correlated genes and query gene set being the genes in each of the two sides (regions) of the module (so for module modAbc in the schematic, two ORA analyses, one for modAbc:BR1 genes and another for modAbc:BR2 genes, are performed). **b** Alluvial plot represents the GO_BP that the modules are enriched for (at a significant cut-off of FDR 5%). Thickness of edges represent the number of modules enriched for each BP for each inter-brain-region comparison. In FP-STG, only query gene sets pertaining to the FP side of the modules are (significantly) enriched. **c** Inter-DC network connectivity between the synaptic signaling annotated genes in mod715 of FP-PHG shown as a heatmap of Inter-DC z-scores. GO_BP annotations of each gene are indicated via color labels (with GO_BP names shortened here; details in Supplementary Fig. 7).

DC genes are expected to provide valuable insights into the underlying biological processes of the clinical development of AD. To identify such biological processes, we partitioned the Inter-DC network into smaller bipartite (two-region) modules using the Louvain algorithm, such that genes within each module are more tightly connected among themselves than with genes in other modules (Fig. 3a). We identified 19–34 modules per inter-brain-region comparison (Supplementary Table 5). In total we obtained 151 modules encompassing 302 gene sets (each module has 2 gene sets, one gene set belonging to each brain region, Supplementary File 2). Enrichment tests for Gene Ontology biological processes (GO_BP, using Over Representation Analysis (ORA); Fig. 3a) showed that most modules are enriched for response to stimulus, synaptic signaling, and synaptic vesicle transporter activities (Fig. 3b). Top GO_BP with the lowest FDR in each module is highlighted in Table 3 (all the enriched functional profiles are in Supplementary File 3 and summarized in Supplementary Fig. 6). Further, we generated random modules, maintaining the same network structure as the identified modules, and did ORA on these random modules—this resulted in a few GO_BP enrichments not related to brain functions, and thereby substantiates the robustness of Inter-DC module enrichments compared to random modules’ enrichment (details in “Methods”).

**Table 3:** Top GO Biological process (GO_BP) with the lowest FDR in each module is highlighted in this table

Brain Region Pair	BR-Module Number	GO_BP Description (id)	Size	Over- Lap	adj. p- value	Genes
FP-STG	FP-mod597	intermediate filament-based process (GO:0045103)	16	3	0.03213037	*INA;NEFM;NEFH*
	FP-mod997	protein targeting to ER (GO:0045047)	28	5	0.00370023	*SPCS1;SSR3;RPLP0;SRP54;RPL41*
	FP-mod1015	synaptic vesicle cycle (GO:0099504)	99	8	0.00246327	*SYN1;STXBP1;RAB3A;SV2A;CHRNB2;DNM1;ITSN1;APBA1*
FP-PHG	FP-mod159	protein folding (GO:0006457)	65	5	4.79E-04	*FKBP4;DNAJA1;HSPA1A;HSPB1;UGGT1*
	FP-**mod715**	synaptic signaling (GO:0099536)	171	21	4.897E-05	*BLOC1S6;YWHAH;GRIN1;RAB3A;PIP5K1C;SYN1;KCNB1;DAGLA;NRGN;SYT1;SHANK2;SYT7;BSN;SLC17A7;SLC12A5;CACNG2;GABBR2;SYT3;SYNGAP1;CACNA1B;IQSEC2*
	PHG-mod488	meiotic chromosome segregation (GO:0045132)	17	4	0.00756791	*BRIP1;NUF2;FANCD2;FANCM*
	PHG-mod250	regulation of cellular response to heat (**GO:1900034**)	18	5	0.00223483	*CHORDC1;HSPA8;HSPA1A;BAG2;FKBP4*
	PHG-**mod715**	neuron projection morphogenesis (GO:0048812)	120	13	0.00142637	*FLRT1;CDK5R1;RAP1GAP;TNR;SYNGAP1;CELSR3;PLAA;SHC3;GRIN1;DNM1L;ULK1;ISLR2;UNC5A*
FP-IFG	FP-mod857	binding of sperm to zona pellucida (GO:0007339)	16	3	0.02900262	*HSPA1L;CCT7;OVGP1*
	FP-mod1005	Translation (GO:0006412)	445	36	0.00376184	*C1QBP;MRPL54;EIF6;MRPS12;MRPL13;MRPS36;SERP1;EIF3I;MRPL14;IARS;NARS;RWDD1;EIF2S1;RNF139;ETF1;MRPS24;MRPL43;MRPL35;UQCC2;LARP6;KARS;RPL32;RPS24;RARS;HARS2;RPL19;MRPS23;RPL21;RPL35A;RPL35;RPL9;MRPS16;RPL36;RPL18;NPM1;TMA7*
	FP-mod1087	detoxification of copper ion (GO:0010273)	11	4	0.00417389	*MT1X;MT2A;MT1E;MT1M*
	FP-mod1189	neuron development (GO:0048666)	685	24	2.4387E-05	*DCDC2;NEFH;NCDN;NEFM;KCNIP2;LIMK1;SPTBN2;ADARB1;DLG4;CNTNAP1;L1CAM;SPTB;SLC12A5;ATCAY;SYT3;FBXO31;NEUROD2;MARK2;INPP5J;CELSR2;PLXNA2;NCKIPSD;ULK1;CPNE5*
	IFG-mod468	ATP synthesis coupled electron transport (GO:0042773)	58	9	0.00025648	*NDUFB3;PARK7;UQCRH;COA6;NDUFA1;NDUFB4;NDUFA8;COX6A1;NDUFC1*
	IFG-mod515	telomere maintenance via telomerase (GO:0007004)	42	7	0.00020626	*CCT8;CCT6A;MAPK1;HSP90AB1;PTGES3;CCT2;NOP10*
	IFG-mod883	regulation of cellular response to heat (**GO:1900034**)	36	5	0.01531659	*CRYAB;HSPH1;HSPA1L;HSPA1A;FKBP4*
	IFG-mod903	chemical homeostasis (GO:0048878)	636	12	0.03676428	*DDB1;THY1;VGF;EPAS1;PQLC2;FABP3;ARF1;ADCYAP1;CHP1;SLC4A4;SYNPO;SLC24A3*
	IFG-mod990	translational initiation (GO:0006413)	120	5	0.00888848	*RPL31;RPL27;EIF6;RPS24;RPL18A*
STG-PHG	STG-mod589	quinone metabolic process (GO:1901661)	8	3	0.00334511	*COQ4;HMGCR;CYP4F11*
	PHG-mod363	response to unfolded protein (GO:0006986)	67	6	0.01006307	*HSPD1;HSPE1;HSPH1;DNAJA1;HSP90AA1;HSPA4*
STG-IFG	STG-mod582	response to heat (GO:0034097)	79	11	0.0002268	*HSPA1A;HSPA6;CHORDC1;DNAJB4;DNAJA1;HSP90AA1;HSBP1L1;PTGES3;DNAJB6;DNAJB1;FKBP4*
	STG-mod1006	response to cytokine (GO:0034097)	539	6	0.03074523	*NMI;FGF2;PLSCR1;HNRNPF;OSMR;IFITM1*
	STG-mod1105	regulation of neuronal synaptic plasticity (GO:0048168)	24	5	0.01499966	*NSMF;SHANK3;SYNPO;JPH3;PPFIA3*
	IFG- mod808	adult walking behavior (GO:0007628)	14	5	0.00179388	*HIPK2;EPHA4;CHD7;OXR1;GLRB*
	IFG-mod1088	regulation of neuronal synaptic plasticity (GO:0048168)	28	10	1.9991E-06	*CAMK2B;DLG4;JPH3;GRIN1;UNC13A;RAB3A;SLC8A2;PPFIA3;CAMK2A;SYNPO*
PHG-IFG	PHG-mod583	cellular macromolecule catabolic process (GO:0044265)	340	16	0.04542191	*RPL15;SKP1;USP47;PSMD1;LSM5;CNOT3;PSMC6;MYEF2;FBXO11;RPL22;FBXL3;TBL1XR1;SET;UBE2K;UBE2D3;ZRANB1*
	PHG-mod770	lymphocyte proliferation (GO:0046651)	91	9	0.0003487	*SYK;PIK3CG;INPP5D;RASAL3;DOCK2;DOCK8;IL18;SASH3;NCKAP1L*

Genes associated with each GO_BP are also noted. A GO_BP and a module name appearing more than once are shown in bold (e.g., mod715).

Among all the modules enriched, the two query gene sets of module number 715 (hereafter referred to as mod715) from each brain region in the FP-PHG pair were enriched for all three GO categories and KEGG pathways, with synaptic signaling being very prevalent. The prevalence of synaptic signaling is also evident from the top ten GO_BPs enriched in the gene sets of mod715 displayed as a dendrogram along with the genes overlapping each BP in Supplementary Fig. 7. To dissect mod715 further, we depicted the DC relation between only the synaptic signaling annotated genes in mod715 in Fig. 3c. *WNK2* (WNK lysine deficient protein kinase 2) from FP and *LZTS1* (Leucine Zipper Tumor Suppressor 1) from PHG have the highest degree in FP and PHG respectively. As evident from ‘The Human Protein Atlas’^37^, both genes and their corresponding proteins are expressed in the cerebral cortex. Studies have shown

*WNK2* is present in cerebral cortex as well as cerebellum of mouse brains, enriched in neurons, and a regulator of GABAergic signaling^38^. Recently, it has been reported that *Lzts1* is associated with microtubule formation, contributes to the increasing intricacy of the cerebral architecture during evolution in mouse, and is mainly enriched in glial cells^39^. Their link to the brain motivates to find their connection with AD pathology. Moreover, we noted that most of the gene-gene correlations are lost in AD compared to CTL in mod715, suggesting that synaptic signaling between FP and PHG is disrupted in AD.

The genes in GO_BP “Synaptic signaling” and its related terms that overlap with the FP vs. PHG side of mod715 are mostly different (Supplementary Fig. 7), thereby reinforcing our previous observation that every brain region’s dysregulated gene sets are distinctive. Only five synaptic signaling related genes, *BSN, CACNA1B, GRIN1, IQSEC2*, and *SYNGAP1*, are shared between the FP vs. PHG gene sets of mod715. *BSN* (Bassoon) is a component of the presynaptic active zone (AZ) involved in organizing the presynaptic cytoskeleton^40^. In contrast, voltage-dependent N-type calcium channel subunit alpha-1B (*CACNA1B*) mediates the ingress of calcium ions (Ca2^+^) into excitable cells, thus controlling the neurotransmitter release from the presynaptic compartment^41^. On the other hand, *GRIN1* encoding the essential subunit GluN1 that is present in all NMDARs (N-methyl-d-aspartate, receptors) found in the postsynaptic membrane, regulates the flow of Ca2^+^ through the channel^42^. Lastly, *IQSEC2* (IQ Motif And Sec7 Domain ArfGEF 2)^43^ and *SYNGAP1* (Synaptic Ras GTPase Activating Protein 1)^44^ form components of the postsynaptic density at excitatory synapses and are critical for the development of cognition and proper synapse function. Excepting *GRIN1*, none of these other genes have been reported till date to be involved in AD pathology^45^. Since all these genes are involved in maintaining synaptic signaling, dysregulation of these genes can lead to neurodegeneration in a variety of disorders, including Alzheimer’s disease. Since our results support involvement of differential correlation of these less-studied genes in AD pathogenesis, it will be interesting to study how the gene and protein expression levels of *BSN*, *CACNA1B*, *IQSEC2*, and *SYNGAP1* are dysregulated in AD, and how that contributes to AD pathology.

In addition to performing ORA with bipartite modules, we conducted ORA for PG, NG, and LC DC edges (mentioned in Supplementary Table 1, 2). A summary of the enrichments is provided in Supplementary Table 6. Noteworthy functions associated with PG-DC edges include cellular organization and transport, synaptic signaling, response to stimuli such as heat and metal ions, and various metabolic pathways including lipid biosynthesis and oxidation-reduction processes. For NG-DC edges, enrichment is observed in regulation of cellular response to stress, protein folding, chaperone-mediated protein folding, DNA metabolic processes, cell cycle regulation, cellular response to heat, and neuronal development. The LC-DC edges are linked to functions such as synaptic plasticity regulation, vesicle-mediated transport, mRNA processing, regulation of cellular localization, and modulation of chemical synaptic transmission. Detailed results from ORA are included in Supplementary File 4.

### AD genetic factors are associated with certain Inter-DC genes and modules

In recent studies on the genetic architecture of AD, a multiplex model has been proposed to understand AD genetics. This multiplex model is based on pathway enrichment analysis using AD risk gene scores^46–48^. Interestingly, we found three of our DC modules, mod715 (FP:FP-PHG), mod770 (PHG:PHG-IFG) and mod1088 (IFG:STG-IFG), to be enriched for GO terms pertinent to certain multiplex model pathways like endocytosis, cholesterol metabolism and immune system process (Supplementary File 5). Specifically, mod715 and mod1088 are enriched for synaptic vesicle endocytosis and presynaptic endocytosis involving genes *DNM1*, *NLGN2*, *PACSIN1*, *PIP5K1C*, *SLC17A7*, *SNCB*, *SYT1* and *SYT7*. Mod1088 is also enriched for ether lipid biosynthetic process and cellular lipid biosynthetic process encompassing genes *AGPS*, *FASN* and *GNPAT*. In addition, mod770 is enriched for different immune system related processes such as T cell proliferation, T cell activation, positive regulation of B cell activation, etc. This prompted us to check if any of the inter-brain-region DC genes and modules we found are linked to AD phenotypes, using results from independent GWAS studies on AD.

Over the last decade, GWAS have revealed many risk loci for AD, implicating many potential causative genes^49,50^ and SNPs (single nucleotide polymorphisms), beyond the well-established APOE association^51^. We test enrichment of such AD risk loci and AD-GWAS signals in the inter-brain-region DC genes/modules, using a tool called MAGMA (Supplementary Fig. 8). Gene-level analysis using MAGMA yielded 208 AD-GWAS genes, i.e., genes for which SNPs in their genomic vicinity are significantly enriched for AD GWAS associations; and 34 of these AD-GWAS genes overlap with the already known AD biomarkers. Out of these 34 genes, *ADAMTS4* is also a DC gene. Further, 125 AD GWAS signal enriched genes out of 208 overlap with DC genes from all six inter-brain-region comparisons. Interestingly our DC hub gene *ZKSCAN1* discussed above is also enriched for AD-GWAS signal, making it an excellent candidate for AD pathogenesis that can be studied further in the context of its DC partners. Other identified AD-GWAS DC genes include *CARF* (Calcium Responsive Transcription Factor) and *PLEKHA1* (Pleckstrin Homology Domain Containing A1), being involved in the rewiring of gene coexpression networks in AD and further based on their functional relevance in the brain, they may also be considered as promising candidates for AD pathogenesis.

Gene set level analysis using MAGMA and multiple testing correction across all the 302 tested gene sets belonging to all 151 modules (identified in the bipartite clustering analysis; see Supplementary Table 5) did not yield any significant result at a stringent cut-off (i.e., none of the modules are significantly enriched for AD GWAS signal at FDR 5%). However as mentioned above, three of our modules are enriched for multiplex model related GO biological processes, which are composed of AD genetic factors. Since disease causal mechanisms have been discovered using disease-associated genetic factors identified in GWAS^52^, our GO enrichment results suggest that these three Inter-DC modules contain risk genes that are causal for AD, besides other genes that are dysregulated as a consequence of AD. To further decipher the molecular factors responsible for inter-brain-region connectivity, we did custom enrichment analysis using signaling molecules.

### Distribution of signaling molecules in inter-brain-region modules leads to molecular hypothesis of AD dysregulation

We inferred the rewiring of the gene network in AD via de-coupling and re-coupling of genes across different brain regions using RNA-seq gene expression data; however the molecular mechanisms supporting this functional organization and re-organization remain elusive. Towards this end, we checked if signaling molecules which are essential for communication between cells or regions (located near or far) overlap significantly with the modules we identified in the rewired bipartite network. Using signaling molecules such as ligand-receptor molecules, Cell-Cell signaling molecules (CCsignaling), CSF markers, secreted proteins (secretome), genes enriched for AD-GWAS signals (208 genes, referred below) and neurotransmitters-neuroreceptors (neurotransmission) as functional categories in ORA, we could uncover 4 signaling/communication-related customized gene sets are enriched in 6 modules after multiple testing correction. (Supplementary Table 7).

Modules from FP-IFG are most enriched for molecules responsible for communication. This may be due to their close proximity as both FP and IFG are located in the frontal cortex. We found 4 customized gene sets namely CCsignaling, CSF, Neurotransmission and Receptor to be enriched. Two modules are enriched for 13 CCsignaling genes and three modules for 34 Neurotransmission genes. Between these two categories, 5 genes namely, *DAGLA*, *IQSEC2*, *RAB3A*, *SYN1* and *SYT1* are found to be common. On the other hand, 11 genes in Receptor category and 3 genes in CSF category are enriched for one module each. It would be interesting to find out which of these signaling molecules are involved in wired (synaptic) transmission and which others in volume (extrasynaptic) transmission.

**Fig. 4:**
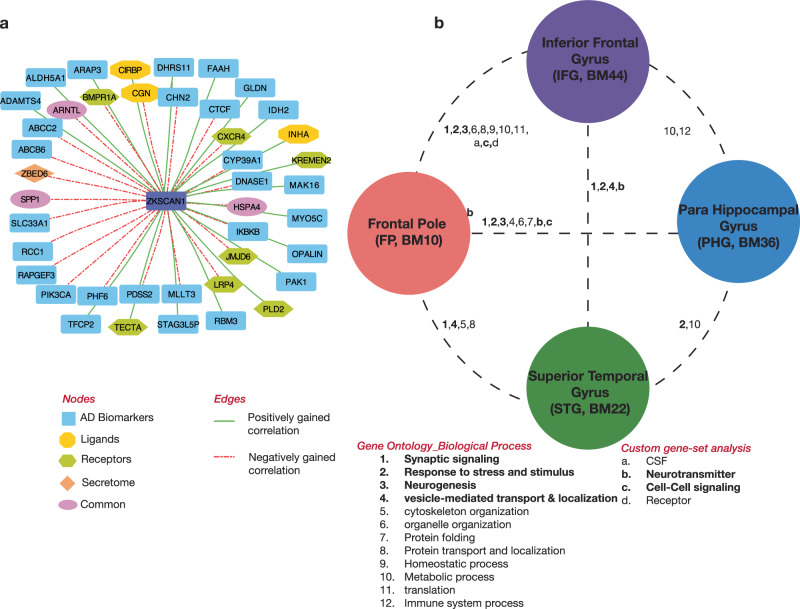
Biomolecules underpinning inter-brain-region communication and disease dysregulation. **a** Inter-DC partners of *ZKSCAN1* are composed of AD biomarkers and signaling molecules. (edges in green solid line represents positively gained edge and in red dashed line represents negatively gained edge, according to the Inter-DC z-score). “Common” denotes those Inter-DC partners that are shared between two nodes. *ARNTL* gene is shared between AD biomarker and secretome, *HSPA4* and *SPP1* genes are shared between AD biomarker and Ligand. **b** Inter(-brain-region)-DC model highlighting the biological processes perturbed in AD due to gene pair rewiring across brain regions. Genes involved in different biological processes that support multiple hypotheses (based on both genetic evidence and clinical trial) of AD pathogenesis are noted in Discussion and those involved in mitochondrial cascade hypothesis are highlighted in Supplementary File 6.

Noteworthy is that the previously determined mod715, which is enriched for synaptic signaling and endocytosis pathway is also enriched for neurotransmission and CCsignaling in the FP side. Interestingly, genes in mod715 is also enriched for the gene ontology molecular function-voltage-gated cation channel activity, particularly calcium ion channel (Supplementary File 5). Voltage-gated cation channel is known to be activated by neurotransmitters. The specific signaling molecules and interactions within the DC module mod715 can thereby lead to specific hypothesis of how this module is affected in multiple regions in AD (Supplementary Table 7). Further, many of the *ZKSCAN1* DC partners are AD biomarkers (34), ligand (5), receptors (7) and secreted proteins (2). This indicates a plausible way how *ZKSCAN1*, by controlling these signaling molecules, (Fig. 4a) enacts its role in AD pathogenesis. Overall this analysis shows the involvement of signaling molecules at different layers of inter-brain-region network.

## Discussion

### An across-region perspective of AD-dysregulated genes and pathways

We have presented here a new DC-based approach to find gene-gene correlations across brain regions that are altered in disease, and use it as a window to inspect how functional coupling among genes in four brain regions can contribute to AD pathology. These analyses enabled us to find how the genic effect of one brain region on another rewires in disease; in this aspect, our study on differential correlation is different from earlier studies on correlations that are conserved between AD and Control groups (e.g., an inter-region study^28^ that showed high number of conserved gene-gene correlations between brain regions connected via AD-associated white matter tracts). We could further decipher genes that are not yet designated as AD biomarkers, but from our analyses, we could clearly observe that they are involved in gene pair rewiring in AD compared to CTL. Partitioning the Inter-DC network into robust modules highlighted that

gene pair rewiring is tightly linked to synaptic signaling and synaptic vesicle transport. Enrichment of these modules further for AD-GWAS signal as well as signaling molecules, helped us to build mechanistic hypotheses supporting the brain molecular connectivity. Further, hub gene analysis revealed *ZKSCAN1* as a key DC gene for most of the inter-brain-region comparisons. Using results from this systematic analysis, we propose an Inter-DC model (Fig. 4b) that gives us a new perspective to decipher the genetic components of AD pathology.

While our approach presents new facts about multiple brain region functioning in AD pathology, it is worth pointing out that our results are limited by the number of brain regions for which data is available; and further should be viewed as *in-silico* genomic-data-driven hypotheses that require further experimental validation, due to the statistical nature (gain/loss of correlation) of the DC relationships. We will get a better view once more genomic data specific for brain regions is available, and experiments are pursued in future around the most promising lead DC relations/genes/pathways from this study to understand the mechanisms leading to Inter-DC. Further, while CTC helps to reduce confounding effects of cellular composition, the cell type (CT) frequencies are estimated only for four major brain cell types and CellCODE cannot also yield absolute cell frequencies. Nevertheless, we get meaningful results that are not enriched for CT specific marker genes. Despite some caveats, the result showing gene-pair rewiring across inter-brain-regions is of much interest and may help to study AD pathology in a new light.

From our analysis, we realized that more than one aspect of synaptic function are affected such as synaptic vesicle trafficking, trans-synaptic signaling, chemical synaptic transmission, and regulation of synaptic plasticity. Interestingly, we noted that even if two brain regions e.g., FP and PHG, are coupled through synaptic signaling, they use a different set of genes to exert their effect. Downregulation of synaptic genes in AD and their specificity to brain regions have been previously reported^53^. However, the usage of different synaptic genes by different brain regions and their functional coupling across brain regions have not been reported before. Shifts in harmony of brain molecular connectivity involving synaptic genes is expected to compromise communication processes between brain regions leading to neurodegeneration, thus causing Alzheimer’s disease.

### Within-region dysregulation in AD

While this study focuses on Inter-DC relations associated with AD, a natural question is the extent to which within-brain-region gene networks are rewired in AD and whether they overlap with the Inter-DC networks. We present results on Intra-DC or Within-DC networks for the four brain regions in Supplementary Table 8. For each brain region BR, we discovered a much higher number of gene pairs that were correlated in the Intra-DC (BR-BR) than Inter-DC (BR-BR2) analysis, due to which many more gene pairs had to be tested for DC. This procedure resulted in subsequently few Intra-DC gene pairs that passed the FDR 1% (adjusted DC *p* value <= 0.01) cutoff, because of high multiple testing burden. To reduce this burden, we performed Intra-DC (BR-BR) analysis on only the gene pairs detected in the Inter-DC networks involving BR (see Supplementary Fig. 4 and Supplementary Table 8 caption). Using this approach, we found that gene pairs involved in Inter-DC relations, when projected onto a single brain region of interest, did not show evidence of Intra-DC within that region (see Supplementary Table 8). Taken together, this implies that Inter-DC rewiring pattern in AD is largely distinct from Intra-DC rewiring.

### Signaling and genetic factors underlying inter-region dysregulation in AD

There remains a lack of detailed mechanistic knowledge about how the brain neuronal network is controlled and how wired transmission and volume transmission complement each other to maintain this brain network. This is complicated by the fact that different brain regions are affected variedly by region specificity as well as AD pathology, adding a spatial element to the disease. However, using the DC framework, we could identify modules of genes, whose gene network architecture between brain regions is altered in AD group relative to controls. We tried to hypothesize and visualize the cause and consequence of gene network dysregulation in AD pathogenesis using these modules of genes. We used customized gene set enrichment analysis to confect our hypothesis. Enrichment of the gene sets “Cell-Cell signaling” and “Neurotransmission” shows that inter-brain-region connectivity is most compromised in AD. Interestingly, mod715 along with synaptic signaling is also enriched for these 2 gene sets. All these analyses help us to hypothesize possible mechanisms around inter-brain-region regulation. It seems during this dysregulation, volume transmission is also compromised making the signaling molecule transport less efficient across brain regions, which may be complementary to Aβ deposition. Recently, spatial transcriptomics studies have shed some light on the spatial element of AD dysregulation, but no such studies have been done for the same pair of regions inspected in our study. When we inspected two AD gene modules from a single-brain-region spatial transcriptomics study (specifically FP-region derived myelin/oligodendrocyte genes; OLIGs and plaque-induced genes; PIGs), we did find those modules to overlap with our Inter-DC genes. But this overlap was not statistically significant, and hence we did not pursue or discuss it further^54^. However as more spatial transcriptomics studies become available in the future, the associated datasets and gene modules will surely help us better understand the overlap between Inter-DC and spatial DE genes.

Finally, hub gene analysis and AD SNP enrichments revealed that *ZKSCAN1*, located in chromosome 7, is a prominent node in the gene network functionally connecting different brain regions and has 309 unique Inter-DC partners. *ZKSCAN1*, when located in IFG, differentially correlates with the largest number of genes in other regions in AD. The pairing of *ZKSCAN1* with genes from different regions is either positively or negatively gained in AD compared to CTL. Previous literature indicates that ZKSCAN1 can act as a transcription factor. Using this information and assuming that ZKSCAN1 protein could be secreted, we looked for a known *ZKSCAN1* motif in the transcription start site of genes pairing with *the ZKSCAN1* gene using the HOMER motif analysis algorithm^55^. However, none of the genes participating in DC relation with *ZKSCAN1* has its corresponding transcription factor motif. This suggests that *ZKSCAN1* uses a different mechanism to maintain correlation with genes from different brain regions (for instance via regulation of target genes within the brain region where ZKSCAN1 is active, and the subsequent effect of these target genes on the genes in the other brain regions), or there is not sufficient statistical power to detect *ZKSCAN1* motifs in its DC partner genes. Conversely, many of the *ZKSCAN1* DC partners are AD biomarkers, ligand, receptors and secreted proteins. This indicates a possible route through which *ZKSCAN1* enacts its function.

### Putting our results in the context of current models for AD pathology/etiology

The complexity of AD can be explained via multiple hypotheses, which are already put through clinical trials. These hypotheses include the cholinergic hypothesis, amyloid hypothesis, tau propagation hypothesis, mitochondrial cascade hypothesis, calcium homeostasis hypothesis, neurovascular hypothesis, inflammatory hypothesis, metal ion hypothesis, and lymphatic system hypothesis^56^. The tau propagation hypothesis and the amyloid hypothesis are believed to interact, and the APOE4 isoform is a significant factor in AD pathogenesis as it affects amyloid-beta (Aβ) clearance and enhances tau hyperphosphorylation. The mitochondrial cascade hypothesis suggests that mitochondrial dysfunction plays a role in AD, impacting the expression and processing of amyloid precursor protein (APP) and the accumulation of Aβ. Mitochondrial dysfunction and oxidative damage play crucial roles in AD, as neurons exhibit increased oxidative stress and reduced mitochondrial numbers. Dysfunctional mitochondria can lead to impaired mitophagy, a process of removing damaged mitochondria, which is regulated by Sirtuins, a class of nicotinamide adenine dinucleotide (NAD)-consuming enzymes that includes nuclear-localized SIRT1, SIRT6, and SIRT7, cytosolic SIRT2, and three mitochondrial SIRTs (SIRT3, SIRT4, and SIRT5). Except SIRT5, all sirtuins specially SIRT4 and SIRT7 are strongly integrated in the DC network (Supplementary File 6). Along with mitophagy, autophagy related genes (ATG) and unc-51-like kinase 1 (ULK1) are also seen to be part of the DC network. Deficiencies in mitophagy and autophagy contribute to AD etiology and may be potential therapeutic targets.

The neurovasculature and inflammatory processes are also implicated in AD. Factors such as hyperlipidemia and obesity increase the risk of AD. Inflammatory cytokines, including tumor necrosis factor (TNF-α) and interleukins, contribute to insulin resistance, Aβ accumulation, and tau phosphorylation. In the Inter-DC network, TNF and interleukins, along with their receptor molecules, have numerous connections such as IL17RB when present in PHG has 111 DC partners in IFG, further emphasizing their involvement in AD. Overall, understanding the interplay between these hypotheses and processes provides valuable insights into the etiology of AD and potential targets for therapeutic interventions, such as enhancing mitophagy and autophagy or utilizing anti-inflammatory drugs to reduce AD occurrence.

Recent studies employing gene set or pathway analysis of AD GWAS signals (using MAGMA or similar methods applied on disease risk scores of genes) have been converging to a multiplex model of AD pathology, wherein multiple pathways are implicated in the genetics of AD. Though an enrichment analysis of our Inter-DC modules for the multiplex model pathways collated from these studies did not yield significant enrichments (which pass a stringent cut-off after multiple-testing correction), our GO enrichment analysis did reveal three of our Inter-DC modules to be significantly enriched for GO terms related to the multiplex model pathways (specifically endocytosis, cholesterol metabolism, and immune related pathways). Our Inter-DC analysis framework thus helps recapitulate certain aspects of the current multiplex-model-based understanding of AD genetics and opens up new avenues to enhance this understanding.

To conclude, comprehending AD pathology is not easy, however understanding brain connectivity alterations can give a better perspective. While functional and structural brain connectomes with respect to AD have been studied for a while now, focus on the molecular basis of these connectomes (molecular connectivity) is rare. Our Inter(-brain-region)-DC framework addresses this gap by enlightening us with new findings and hypotheses on how AD affects the coupling between genes and biological processes in different brain regions, mediated by signaling molecules that aid in synaptic (wired) or extra synaptic (volume) transmission. These results demonstrate the value of inter-brain-region analysis in AD, and encourage its application to different neurological diseases and extension to inter-organ/inter-tissue analysis to understand the molecular connectome of the whole body.

## Methods

### Data collection

The data was collected and pre-processed as per original study and also used in our recent study Multicens, to address different sets of questions compared to this study. The covariate-adjusted RNA-seq data with the following synapse ids - syn16795931 – Brodmann Area (BM10) – frontal pole (FP), syn16795934 - BM22 - superior temporal gyrus (STG), syn16795937 - BM36 - parahippocampal gyrus (PHG), syn16795940 – BM44 - inferior frontal gyrus (IFG), were downloaded from AD Knowledge Portal – The Mount Sinai/JJ Peters VA Medical Center Brain Bank cohort (MSBB) study (10.7303/syn3159438). The pre-processed data is corrected for library size differences using the trimmed mean of M-values normalization (TMM method – edge R package) and linearly corrected for sex, race, age, RIN (RNA Integrity Number), PMI (Post-Mortem Interval), sequencing batch, exonic rate and rRNA (ribosomal RNA) rate. As in the earlier study^26^, normalization was performed on the concatenated data from all four brain regions to avoid any artificial regional difference.

The clinical (MSBB_clinical.csv) and experimental metadata (MSBB_RNAseq_covariates_November2018Update.csv) files available on the portal are used to classify the samples into control (CTL) and Alzheimer’s disease (AD) based on CERAD score (Consortium to Establish a Registry for AD; funded by NIA, 1986)^57^. CERAD score 1 was used to define CTL samples, and 2 (‘Definite AD’) was used for defining AD samples. Probable AD (CERAD = 3) and Possible AD (CERAD = 4) samples were not considered for this study. Sample sizes divided according to the four brain regions along with metadata are noted in Supplementary Table 9. Further, we considered two brain regions at a time for our analyses and selected the samples accordingly to handle missing data (Supplementary Table 10; note that not all individuals had all four brain regions sampled).

The genes in the gene expression data are denoted in the hg37 ENSEMBL gene identifier (ENS. ID) format. The initial analysis is performed using the ENS. ID. For the downstream analyses (visualization/enrichment), the ENS. IDs are mapped to the HGNC gene symbols using the comprehensive gene annotation file for Release 19 (GRCh37.p13) downloaded from Gencode - https://www.gencodegenes.org/human/release_19.html (h37).

The Wang et al., 2016 study^27^ ranked 19 brain regions for their vulnerability to AD based on how many genes in these regions are associated with disease status (DE genes) and disease traits like the accumulation of NFT and Aβ. The brain regions used in this study are sorted based on the same ranking, such as BM36: rank 1, BM44: rank 2, BM22: rank 7, and BM10: rank 14, with rank 1 being the most vulnerable region in AD, and other ranks being proportionately less vulnerable.

To perform replication testing, we retrieved data on an independent cohort associated with the Harvard Brain Tissue Resource Center (HBTRC)^58^. Two brain regions - visual cortex (VC, BM17) and dorsolateral prefrontal cortex (DLPFC, BM9) - comprising of 300 samples (116 CTL and 184 AD) was used for our study. Gene expression data has been linearly adjusted for these covariates: age, gender, RIN, Batch, PMI and pH. Missing value of any covariate has been imputed with the respective mean value. Adjusted data is subjected to CTC as is done for MSBB; and same protocol for DC analysis is followed in this case also (explained below).

### Cell type correction (CTC)

The expression of a gene in a bulk tissue can be captured by the proportion of different cell types in the tissue and the expression of the gene in these cell types^59,60^. Our ideal aim is to remove the former contribution and study the latter to reveal cell-intrinsic changes in gene pair correlation structure between disease vs. control group. Towards this, we corrected the bulk gene expression data for cell-type proportions, which were in turn estimated from bulk data using a cellular deconvolution method. Specifically, we estimated the frequencies of four major brain cell types, astrocytes, microglia, neuron, and oligodendrocytes, using a cellular deconvolution method implemented in the getAllSPVs function from the CellCODE (Cell-type Computational Differential Estimation) R package^61^. CellCODE is a singular value decomposition (SVD) based reference-free method to perform cellular deconvolution. It only requires the RNA-seq expression matrix of a set of marker genes. Human marker genes (markers_df_human_brain data frame) for the four major cell-types were obtained from the BRETIGEA (BRain cEll Type specIfic Gene Expression Analysis) meta-analysis study^62^. CellCODE performs F-tests on the supplied set of marker gene expression data to identify robust marker genes i.e., marker genes, which are not differentially expressed between the disease group vs. control groups. Only these robust marker genes are used to estimate cell-type proportions. The cell-type corrected gene expression data is obtained by linearly adjusting the bulk RNA-seq data for the cell-type proportions estimated using CellCODE.

Patrick et al. (2020) study^60^ generated gene expression and Immunohistochemistry (IHC) data. We use the cell-type proportions for neuronal, astrocyte, microglia, and oligodendrocyte cells from this study to assess the performance of our cellular deconvolution methods. Data is available at https://github.com/ellispatrick/CortexCellDeconv. We compared the cell-type estimates obtained for MSBB BM10 brain region with the IHC estimates, since BM10 is the closest brain region when considering the brain tissue from which IHC data was generated. We evaluated two different methods of cellular deconvolution, namely BRETIGEA and CellCODE on the cortical gene expression data set from Patrick et al. 2020^60^ to identify the best performing model, i.e., the model with the highest correlation with the IHC estimated cell-type proportions – which can be considered the ground truth data. For BRETIGEA, we used the function call: brainCells(geneExpmatrix, nMarker = 20, species = “human”), where nMarker is the number of markers that will be considered for each cell type to build the model. For CellCODE, we used the getAllSPVs function with input arguments: data, dataTag, grp, method, and mix.par, to build the model. Data is the gene expression data of the marker genes, dataTag is a binary matrix (# marker genes (MG) X # cell-types) which indicates which marker genes are associated with which cell type, and grp is the CERAD classification of each sample considered. Mixed method at the CellCODE-suggested 0.3 mix.par cutoff was used. The models for BRETIGEA and CellCODE were built using different sets of top 20, 40, 80, 200, 500, and 1000 marker genes sets for each of the four cell types to arrive at the best model.

Through this analysis, CellCODE 80 MG (i.e, 20 MG each of the four major cell types) was identified as the best performing model for predicting neuronal frequencies and henceforth used to estimate the relative frequencies of the other cell types as well for consistency. These predicted cell frequencies could in turn be used for the CTC. Specifically, using CellCODE, we built one cellular deconvolution model for each brain tissue. By inferring the DC interactions from the CTC data, which is corrected for the cell-type composition effects, the confounding influence of cell-type proportions on the DC results is mitigated^63,64^. To underscore the significance of cell type correction (CTC) and its impact on our DC analysis, we have provided a comparison of DC edges before and after applying CTC in Supplementary Table 11. The Table shows that CTC causes a significant reduction in the number of DC edges identified at FDR 1%. Nevertheless, there is still a sufficient number of DC pairs that we had identified from the CTC data, and we are more confident about these pairs being driven by cell-intrinsic DC signal (the focus of our study), rather than cellular composition effects. The final set of DC pairs includes pairs that are originally masked by cellular composition effects but revealed after CTC, and also DC pairs that are resilient to cellular composition effects (and hence found in both before- and after-CTC analyses; see Supplementary Table 11).

### Differential correlation (DC) analysis

We are interested in identifying gene pairs across brain regions whose correlation strength in the disease group (AD) is significantly different from that in the control group of individuals (CTL), and call such pairs as differentially correlated or co-expressed (DC) pairs. We also use the terms *DC* and *Inter-DC* interchangeably in this work, since these DC pairs that we work with represent inter-brain-region DC gene pairs.

Gene-gene spearman correlation coefficients (ρ) for each of the gene pair combinations possible across brain regions are calculated for the AD group and CTL group separately. The Spearman correlation p-values are corrected for multiple testing using the Benjamini-Hochberg (BH) FDR method, and the resulting BH-corrected p-values are subject to a 1% FDR cutoff to identify statistically significant correlation coefficients. All gene pairs significantly correlated either in the AD or Control group are considered for the Inter-DC analysis^65^. Here, absolute correlation cut-off of at least 0.4, moderate strength, is used to call a gene pair correlated. Note that we are not considering gene-gene interaction within a particular tissue. The union of correlated gene pairs of AD and CTL groups for any inter-brain-region comparison is referred to as *correlated pairs* throughout the manuscript. Only these correlated pairs are tested for DC.

We use the r.test function from the psych R package to test a gene pair for DC. The r.test function transforms the AD as well as CTL gene-gene correlation coefficient values obtained for each gene pair into their corresponding z scores, known as the Fisher’s r to z transformation. The difference between the Fisher z transformed correlation coefficients, divided by the standard error of the difference, yields the final z-scores and associated DC p-values to be tested. For any inter-brain-region comparison BR1-BR2 (Brain Region 1–2), we subject the DC *p*-values of all correlated gene pairs in BR1-BR2 to multiple testing correction using the Benjamini-Hochberg FDR method and use 1% FDR cut-off to report significant DC pairs. For any given inter-brain-region comparison, the DC Dsyregulation Index is the ratio of the number of significant DC gene pairs detected for that region pair to the number of all gene pairs tested for DC (i.e., all correlated pairs) for the same region pair. Note that the sign of a (Inter-DC) z-score indicates whether a particular DC gene pair’s correlation coefficient increased (positive z score) or decreased (negative z score) in the AD group relative to the CTL samples.

In addition, to check whether the sets of DC gene pairs in two inter-brain-region comparisons are similar, the Jaccard similarity index, which is the ratio of the intersection of two sets to the size of their union, was calculated^66^.

We tested DC gene pairs found in inter-brain region analysis from MSBB cohort for replication using another cohort data from HBTRC, where different brain regions have been profiled using different technology (microarray)^58^. This replication test has lent some confidence to proceed with further downstream analyses and reinforced the robustness of our methodology and findings (Supplementary Fig. 9).

### Differential gene expression analysis

In this study, Differentially Expressed Genes (DEGs) were identified from CTC (cell type corrected) bulk RNA-seq data using a Wilcoxon rank-sum test for each of the four brain regions. DEGs identified at FDR cut-off 0.05, 0.1, and 0.2 were used to check whether the Inter-DC relation between each gene pair is driven by DEGs or not.

### Identification of bi-partite (two-region) modules

The set of gene pairs identified as DC for two given brain regions (BR1 and BR2) can be viewed as a bipartite (two-layered) network of Inter-DC relations. We are interested in identifying a module comprising one set of genes in the first region (BR1) and another in the second region (BR2) that participates in many DC relations among themselves. We would also prefer that the modules be tightly-knit modules such that genes within a module are more likely to be related to one another than they are to the rest of the network. These preferences can be expressed as a modularity objective function. The bipartite network can be partitioned into a collection of modules that maximize this modularity function using a heuristic method called the Louvain method^67^. The cluster_louvain function under the R package igraph was used for this purpose. Using the ‘modularity’ function, we calculated the modularity score for each bipartite network (inter-brain-region DC gene set). To detect the modules enriched for significant Gene Ontology (GO) biological categories and pathways, we set a threshold that at least 20 genes must be present in each module. Partitioning each DC gene pair list from each inter-brain-region comparison resulted in multiple modules (see Supplementary Table 5). Each module comprises two gene sets, one from BR1 and one from BR2.

### Over representation analysis (ORA)

Over Representation Analysis (ORA) is a method that tests if genes from pre-defined functional sets (such as those belonging to a specific GO term or KEGG pathway) are enriched or over-represented (i.e., present more than would be expected by chance) in a given query set of gene. To identify the potential biological functions associated with the gene sets in the modules we identified, we performed ORA using the WebGestaltR package^68^. Biological processes and pathways are controlled by vast interacting molecules whose expression levels are frequently co-regulated or co-expressed. After identifying tightly correlated Inter-DC modules, we performed over-representation analysis (ORA) to test if a set of DC genes is enriched for genes belonging to known Gene Ontology (GO) categories. We performed this enrichment analysis only on modules of reasonable size (specifically those with at least 20 genes). Each module consists of 2 gene sets, one from each brain region (BR1 or BR2). A correlated gene list corresponding to each brain region (union of AD group and CTL group) was used as the background genes for this analysis, whereas the DC genes from each module acted as query genes. WebGestaltRBatch function was used to run the enrichment analysis so that the gene sets for multiple modules can be submitted at the same time. Under the ‘Functional database category’, Gene Ontology, GO (Biological process, cellular component, and molecular function), and pathways (KEGG & REACTOME) were selected for enrichment. We used FDR thresholds of 0.05 and used redundancy reduction methods (affinity propagation and weighted set cover) to find the most significantly enriched terms. In the main manuscript mainly GO_BP results are highlighted. Detailed GO_BP result is included in Supplementary File. 5 whereas the rest of the functional enrichment results (molecular function, cellular components, KEGG and Reactome pathways) are included in Supplementary File 3.

For the enriched modules, we ran the ORA with ShinyGO v0.66^69^ to generate the hierarchical clustering tree. This tree groups related GO terms together based on how many genes they share. The top 10 processes were selected for hierarchical clustering tree representation.

We also used customized functional categories, including genes enriched for AD GWAS signal, ligand-receptor molecules, CCsignaling, CSF markers, secretome, and neurotransmitters-neuroreceptors (neurotransmission) for ORA. The AD GWAS enriched genes are retrieved from MAGMA analysis (explained below). Ligand-Receptor pairs are assembled by combining the latest data of the year 2020 from GitHub repositories (https://github.com/LewisLabUCSD/Ligand-Receptor-Pairs). CSF markers are extracted from literature mining. Secreted proteins, CCsignaling and neurotransmitters are downloaded using AmiGo^70^. Custom gene sets as .gmt file is available in Supplementary File 7.

#### Robustness check for ORA

We wanted to check if the enrichment of Inter-DC modules for GO Biological Processes or other functional categories/pathways are statistically significant, compared to enrichments seen in some random modules. For that we generated random modules from the correlated gene list for each brain region per inter-brain-region comparison, maintaining the respective module structure based on DC module identifiers. For each inter-brain-region pair, using Louvain algorithm we generated Inter-DC modules, each gene being designated with module id. We used the same module id list for the respective inter-brain-region pair and background genes, sampled it every time and generated the random modules based on the sampled module id. In total, 10 permutations are done for each region per inter-brain-region comparison. Total number of modules enriched in DC vs. random is represented in Supplementary Table 12 and Supplementary Fig. 10a, 9b. Further, in Supplementary Fig. 10c, 9d, empirical FDR of 2 representative modules reflects the robustness of DC module enrichment. Lastly, Supplementary Table 13 highlights that the functions enriched in random module are not related to brain or AD pathology except for one GO_BP “GO:0007628- adult walking behavior”. This clearly reflects Inter-DC module enrichment is much more robust and meaningful compared to random modules’ enrichment.

In the case of custom gene set enrichment, we retrieved the raw p-values of all the 302 gene sets (151 modules) tested and adjusted them to perform multiple testing correction using the Benjamini-Hochberg FDR method (implemented in p.adjust function in the R programming environment). Only those that are enriched at FDR < = 0.05 cut-off are reported to be enriched (Supplementary Table 6). Same adjustment was done for testing multiplex model pathway enrichment in our Inter-DC modules.

### SNP enrichment analysis

GWAS studies have revealed numerous risk loci associated with AD, which harbor putative causative genes and variants. We aimed to check if Inter-DC genes or Inter-DC module gene sets are enriched for such GWAS-detected AD associations. The AD GWAS association signals in the form of SNP summary statistics are available for a comprehensive set of SNPs from a recent meta-analysis study of four major AD GWAS studies - the Psychiatric Genomics Consortium (PGC-ALZ), the International Genomics of Alzheimer’s Project (IGAP), the Alzheimer’s Disease Sequencing Project (ADSP), and UK Biobank (UKB). This study assessed the effect of 9,862,738 SNPs in 71,880 AD samples and 383,378 controls samples^11^. We would now like to test whether a given gene (or set of genes) is in the vicinity of many SNPs associated with AD in the above meta-analysis study. For this purpose, we use MAGMA, a tool for gene analysis and generalized gene-set analysis of GWAS data, in order to predict gene and gene-set level p-values using SNP-level *p* values^71^. Inputs to MAGMA include SNP summary statistics of the meta-analysis study^11^ (downloaded from the CNCR/CTG LAB (Center for Neurogenomics and Cognitive Research/Complex Trait Genetics) website), and European 1000 Genomes reference data as described next. 22,665,064 SNPs retrieved from European 1000 Genomes data files were first annotated to 19,354 genes from the hg19 genetic reference (human genome Build 37), using a 10 kb annotation window on either side of the gene. Next, using SNP *p* value and European 1000 Genomes reference data, 18,445 genes were mapped to SNPs, of which genes significantly enriched for AD GWAS signal at FDR 5% (BH-adjusted *p* < 0.05) were retained. Further, MAGMA basic gene set analysis was performed on 302 gene sets (151 Inter-DC modules), to test if these gene sets were significantly enriched for AD GWAS signal at FDR 5% (BH-adjusted *p* < 0.05).

### Reporting summary

Further information on research design is available in the Nature Research Reporting Summary linked to this article.

### Supplementary information

Supplementary Information

Reporting Summary

Suppl File 1: Inter-brain-region DC gene pair interactions- All DC gene pairs in six inter-brain-region comparisons at FDR 1% are noted. The first sheet contains the header description. From the second sheet onwards all the 6 inter-brain-region comparisons are included, in 6 separate sheets. The last sheet contains the top 20 DC hub genes from 6 Inter-DC networks.

Suppl File 2: Module details- 302 Genes sets (151 modules) are included in this .gmt file. The first column contains the gene set name [e.g., 1015BR1BM10-22 means module 1015 belongs to brain region 1, BR1 (BM10 or FP in this case) in the inter-brain-region comparison BM10-22 or FP-STG]. The second column in a .gmt file is meant for description. No description is included, hence is denoted with “na”. From the third column onwards all the gene entrez ids for the respective gene set are noted.

Suppl File 3: Functional Enrichment- Enriched functional profiles of DC modules. Gene Ontology Molecular Functions & Cellular Components and KEGG & Reactome pathways are represented in the functional profile of each module. The first sheet contains the legend, and the second sheet has a summary of the number of modules enriched for each category & the parameter description. From the third sheet onwards results for all the 6 inter-brain-region comparisons are included, in 6 separate sheets.

Suppl File 4: GO_BP Enrichment of GOC/LOC DC edges- Enriched Gene Ontology Biological Processes (GO_BP) for PG, NG and LC DC edges are noted.

Suppl File 5: GO_BP Enrichment- Enriched Gene Ontology Biological Processes (GO_BP) are represented. The first sheet contains the legend and the parameter description. The second sheet contains all the module enrichment results.

Suppl File 6: Genes involved in Mitochondrial cascade hypothesis- This file summarizes the DC interactions in each brain region pair involving different Sirtuins [SIRTs] genes, which helps in mitophagy regulation.

Suppl File 7: Customized gene sets- This .gmt file contains the 10 signaling based functions along with the genes associated for each term. The first column contains the gene set name [e.g., SNP, neurotransmission, cell-cell signaling etc.]. The second column in a .gmt file is meant for description. No description is included, hence is denoted with “na”. From third column onwards all the gene symbols for the respective gene set are noted.

## Data Availability

The primary data pertaining to MSBB cohort analyzed in this study has been previously published and available via the AD Knowledge Portal (as described in detail in Methods). Availability of all other data used in this study has also been described in the main text and associated Supplementary Information files.

## Code availability

The source code relevant for conducting the analyses in this study and pointers to the associated data/results are available at https://github.com/BIRDSgroup/InterTissueDC.
