# Supplementary material for: Alzheimer’s disease rewires gene coexpression networks coupling different brain regions
Source: NPJ Syst Biol Appl. 2024 May 9;10:50. doi: 10.1038/s41540-024-00376-y (PMC11082197; doi:10.1038/s41540-024-00376-y)
Supplement: Supplementary file 1 — Supplementary Information [file 41540_2024_376_MOESM1_ESM.pdf]

Alzheimer's disease rewires gene co-expression networks coupling different brain regions

Supplementary Information

Supplementary Tables

Supplementary Table 1. Classes of DC pairs detected by our analysis. For a DC gene pair, its class is based on the gene pair's correlation in the CTL and in the AD group (1 means significantly positively correlated, -1 means significantly negatively correlated, and 0 means neither; see Methods for cut-off used to determine significant correlation).

LC= Lost Correlation
NG= Negatively gained correlation in AD
PG= Positively Gained correlation in AD

CTL	AD	Status/Class	DC z-score
0	1	PG	Positive
0	-1	NG	Negative
1	0	LC	Negative
-1	0	LC	Positive

Supplementary Table 2. For each inter-brain-region comparison, the number of DC gene pairs are divided into gained positive & negative correlation and lost correlation as described in Fig. 1c. Abbreviations of PG, NG and LC explained in Suppl. Table 1.

Brain Region Pairs	CTC DC	PG	NG	LC
FP-STG	2961	1059	1109	793
FP-PHG	2629	844	824	961
FP-IFG	9962	4512	4899	551
STG-PHG	6274	2971	3082	221
STG-IFG	8179	3265	3361	1553
PHG-IFG	12979	5953	6174	852

Supplementary Table 3. Summary of the common genes shared by two regions in an inter-region comparison. Gene neighbours of these common genes are also inspected for overlap. Identical gene pairs per comparison are also noted.

Brain Region Pairs (BR1-BR2)	Total DC edges	BR1 DC genes	BR2 DC genes	Common Genes	Gene neighbours of common genes (DC edges)		Common neighbour genes	Identical gene pairs
					BR1	BR2		
FP-STG	2961	2013	1844	260	354 (444)	392 (472)	26	1
FP-PHG	2629	1597	1409	261	387 (521)	439 (606)	46	14
FP-IFG	9962	4609	4235	1299	1795 (3392)	1850 (3338)	314	12
STG-PHG	6274	2580	2549	754	1071 (2595)	1229 (3023)	356	18
STG-IFG	8179	3297	3548	968	1520 (3198)	1409 (2932)	287	14
PHG-IFG	12979	3642	4202	1348	2441 (7402)	1780 (6279)	624	31

*Note- BR1 and BR2 represents brain region 1 & 2 respectively for each inter-region comparison. For example, in FP-STG brain region pair, FP is BR1 and STG is BR2.This abbreviation is used in subsequent tables also. In the case of FP-STG, there are 260 genes common between 2 regions, which is 13% of 2013 (FP) and 14% of 1844 (STG) genes. These common genes have 472 connections (DC edges) from BR1 to BR2 and 444 from BR2 to BR1, i.e., 260 genes of BR1 interact with 392 genes in BR2. Among these gene neighbours, only 26 genes are common. Further, 1 gene which forms identical gene pair is basically a subset of 260 common & also 26 common neighbour genes.

Supplementary Table 4. Hub genes along with their degree in each inter-region comparison. Degree of a gene refers to the number of its DC partners (for abbreviations, refer to the note above).

**Brain Region Pairs (BR1-BR2)**	**BR1**			**BR2**		
	**Highest Degree**	**Gene Symbol**	**Gene Name**	**Highest Degree**	**Gene Symbol**	**Gene Name**
FP-STG	20	*RP11-418J17.1*	WARS2 antisense RNA 1	35	*NTM*	Neurotrimin
FP-PHG	41	*FAM86B3P*	Family With Sequence Similarity 86 Member B3, Pseudogene	42	*LZTS1*	Leucine Zipper Tumor Suppressor 1
FP-IFG	61	*TTLL7-IT1*	lncRNA: TTLL7 Intronic Transcript 1	113	*CACYBPP1*	Calcyclin Binding Protein Pseudogene 1
STG-PHG	42	*PPDPF*	Pancreatic Progenitor Cell Differentiation And Proliferation Factor	81	*FAM86B3P*	Family With Sequence Similarity 86 Member B3, Pseudogene
STG-IFG	48	*FSD1*	Fibronectin Type III And SPRY Domain Containing 1	86	*PLK3*	Polo Like Kinase 3
PHG-IFG	111	*IL17RB*	Interleukin 17 Receptor B	82	*ZKSCAN1*	Zinc Finger With KRAB And SCAN Domains

Supplementary Table 5. Statistics of modules detected in each brain inter-region comparison. Largest module size (number of genes) for each comparison is noted. The number of modules used for enrichment analysis, based on a minimum size of 20 genes, is also shown.

**Brain Region Pairs**	**Total # Modules**	**Max Size**	**Module Size >= 20**
FP-STG	1051	105	19
FP-PHG	737	225	19
FP-IFG	1235	651	34
STG-PHG	907	313	27
STG-IFG	1130	424	29
PHG-IFG	1057	766	23

Supplementary Table 6. Top GO Biological process (GO_BP) with the lowest p-value in each brain region pair is highlighted in this table.
Abbreviations of PG, NG and LC explained in Suppl. Table 1.
GOC= Gain of correlation; LOC= Loss of correlation

BR Pair	BR	GOC/LOC	description	size	overlap	FDR
FP-STG	FP	LC	synapse organization	384	24	0.01863291
FP-PHG	FP	LC	regulation of cellular localization	867	47	0.00037333
FP-PHG	PHG	LC	neuron development	1064	39	0.00405329
STG-PHG	PHG	LC	SRP-dependent cotranslational protein targeting to membrane	95	7	0.0404381
STG-IFG	IFG	LC	ribonucleoprotein complex subunit organization	245	32	6.3313E-05
STG-IFG	STG	LC	intracellular transport	1788	107	0.00015752
PHG-IFG	IFG	LC	protein modification by small protein conjugation or removal	1004	51	0.00016572
FP-PHG	FP	NG	regulation of B cell differentiation	27	7	0.01137434
FP-PHG	PHG	NG	cellular response to heat	98	12	0.00074213
FP-IFG	FP	NG	cellular protein localization	1805	257	0.00318875
FP-IFG	IFG	NG	cellular response to stress	1849	248	3.6505E-06
STG-PHG	FP	NG	cellular amino acid metabolic process	297	45	8.6922E-05
STG-PHG	PHG	NG	regulation of cellular response to heat	44	14	0.00026107
STG-IFG	IFG	NG	response to endogenous stimulus	1585	154	0.00241727
STG-IFG	STG	NG	cellular response to stress	1849	175	0.00039453
PHG-IFG	IFG	NG	plasma membrane bounded cell projection organization	1457	211	0.00058778
PHG-IFG	PHG	NG	cellular response to stress	1849	187	0.00336126
FP-PHG	FP	PG	chromosome organization	1088	43	0.02129853
FP-IFG	FP	PG	trans-synaptic signaling	691	103	0.00809093
FP-IFG	IFG	PG	intracellular transport	1788	239	3.3775E-05
STG-PHG	PHG	PG	response to topologically incorrect protein	187	27	0.02061273
STG-IFG	IFG	PG	mitotic cell cycle	910	101	0.00342387
STG-IFG	STG	PG	cell cycle	1716	157	0.00220188
PHG-IFG	IFG	PG	plasma membrane bounded cell projection organization	1457	206	0.00018947

Supplementary Table 7. Customized enrichment. Modules enriched using customized set via ORA are listed below. The brain region and the respective brain region pair to which a module belongs is designated in the first column. E.g., mod715 belongs to Frontal Pole (FP) side of brain region pair FP-PHG. Size of the customized gene set and overlap with a particular module are also included along with the overlapping gene names and adjusted p value (BH method= Benjamini and Hochberg method).

**BR:BR Pairs**	**Module Name**	**Customized Gene Set**	**Size**	**Overlap**	**Overlapping genes**	**BH**
FP:FP-PHG	mod715	CCsignaling	299	10	*RAB3A;SYN1; BSN;KCNB1; DAGLA;SYT1; SHANK2; GABBR2; CACNA1B; IQSEC2*	0.031493
FP:FP-IFG	mod857	CCsignaling	299	3	*GRM3;GRIA2; HSPA8*	0.048592
IFG:FP-IFG	mod903	CSF	131	3	*VGF;FABP3; GAP43*	0.048592
IFG:STG-IFG	mod1088	Neurotransmission	469	24	*CLSTN1; FRRS1L; SYT7;CHRM1; NRXN2;DLG4; NLGN2; DLGAP3; IQSEC2; STXBP1; GRIN1;EPS8; DLGAP4; RIMS4; DLGAP2; LYNX1; PIP5K1C; UNC13A; RAB3A; GRIK5;PPFIA3; SLC17A7; SLC6A7; CAMK2A*	1.16E-05
FP:FP-PHG	mod715	Neurotransmission	469	12	*BLOC1S6; GRIN1; RAB3A;*	0.031493

					PIP5K1C; SYN1; DAGLA; DLGAP2; SYT1; SYT7; SLC17A7; CACNG2; IQSEC2	
STG:STG-IFG	mod1105	Neurotransmission	469	9	*SHANK3; MAPK8IP2; RIMS4; BRSK1; DLGAP3; SYNGR3; SHANK1; DAGLA; PPFIA3*	0.031493
FP:FP-IFG	mod756	Receptor	919	11	*CD86; FCGR2A; INSR;GLRA2; FLOT1;RGMB; TECTA;IGF2R; KCND2; SLC44A5; PLXNA4*	0.048592

Supplementary Table 8. **Statistics on gene pairs tested for within/intra-region DC (also known as Intra-DC).** For each brain region BR, two sets of gene pairs were tested for Intra-DC: (i) All gene pairs correlated either in AD or Control group, following the same protocol as for Inter-DC. (ii) A subset of the correlated gene pairs above that have a corresponding Inter-DC edge centered at the brain region of interest. In each of the above case, the DC p-values of the tested gene pairs were corrected for multiple testing and the resulting Intra-DC edges discovered at FDR 1% are reported.

Brain Region (BR)	# tested for Intra-DC (i)	# discovered for Intra-DC (i)	Inter-DC centered at BR	# tested for Intra-DC (ii)	# discovered for Intra-DC (ii)	Fraction discovered for Intra-DC (ii) (%)
FP	32,994,204	8	15498	8108	86	1.1
STG	10,052,729	1	17323	8193	25	0.3
PHG	24,005,107	50	21587	11947	148	1.2
IFG	16,182,597	4	30762	17299	684	4

Supplementary Table 9. Sample sizes in the four brain regions along with adjusted metadata.

Brain Regions	Group	No. of Samples	Gender		Mean (SD)		
			Male	Female	AOD	PMI (minutes)	RIN
FP	CTL	75	35	40	81.27 (8.19)	563.07 (391.56)	6.74 (1.49)
	AD	111	42	69	83.33 (7.42)	379.14 (254.56)	6.40 (1.45)
STG	CTL	65	31	34	81.12 (8.61)	574.26 (397.42)	6.68 (1.49)
	AD	101	42	59	82.62 (7.53)	371.99 (264.59)	6.45 (1.50)
PHG	CTL	68	33	35	80.81 (8.36)	546.49 (395.02)	6.87 (1.36)
	AD	90	33	57	83.58 (7.3)	365.42 (262.38)	6.54 (1.47)
IFG	CTL	64	32	32	81.11 (8.5)	572.83 (402.95)	6.80 (1.57)
	AD	90	34	56	83.74 (7.39)	375.26 (267.56)	6.59 (1.48)

*AOD - Age of Death, PMI - Post Mortem Interval

Supplementary Table 10. Sample sizes in the inter-brain region comparisons.

Brain Region Pairs	CTL	AD
FP-STG	56	88
FP-PHG	57	76
FP-IFG	58	79
STG-PHG	55	73
STG-IFG	53	79
PHG-IFG	54	68

Supplementary Table 11. The number of DC gene pairs (edges) identified from cell type corrected (CTC) and non-cell type corrected data (noCTC) data.

Brain Region Pairs	CTC DC	noCTC DC	Common DC pairs
FP-STG	2961	17681	362
FP-PHG	2629	20185	395
FP-IFG	9962	12210	1056
STG-PHG	6274	19000	455
STG-IFG	8179	346050	1585
PHG-IFG	12979	27584	1710

Supplementary Table 12. DC vs Random module enrichment statistics
For random modules, total number of modules enriched is divided by 10 as we have done 10 permutations.

	# Modules	# Module * GO_BP
**DC**	25	416
**Random**	3.1	14

stands for "Number of"

Supplementary Table 13. GO_BP enriched in Random modules.
This table clearly represents that for random modules, functions (GO_BP) enriched are not related to processes in brain or AD pathology. Top GO_BP (with lowest FDR) for each brain region per inter-region comparison is noted in this table.

**Brain Region Pair**	**Brain Region**	**GO id**	**description**	**size**	**overlap**	**FDR**	**Genes**
FP-STG	FP	GO:0035338	long-chain fatty-acyl-CoA biosynthetic process	9	3	0.03042	*HSD17B12;ACSL1;ELOVL7*
FP-STG	STG	GO:0060445	branching involved in salivary gland morphogenesis	11	3	0.02147	*BTBD7;LAMA5;FGFR1*
FP-PHG	PHG	GO:0000055	ribosomal large subunit export from nucleus	5	2	0.02309	*NUP88;RAN*
FP-IFG	FP	GO:0007628	adult walking behavior	17	3	0.02063	*GLRB;HTRA2;CEND1*
FP-IFG	IFG	GO:2000114	regulation of establishment of cell polarity	15	3	0.03089	*ROCK2;WDPCP;KIF20B*
STG-PHG	STG	GO:0070203	regulation of establishment of protein localization to telomere	5	2	0.02094	*CCT3;CCT5*
STG-PHG	PHG	GO:0042255	ribosome assembly	11	4	0.00966	*NOP2;RPL5;RPL12;RPS28*
STG-IFG	STG	GO:2001235	positive regulation of apoptotic signaling pathway	90	7	0.00371	*G0S2;NCK2;TRAF2;AGT;DDIT3;BCL2;INHBA*

PHG-IFG	PHG	GO:0032494	response to peptidoglycan	10	4	0.00106	*C5AR1;IRF5;CARD9;RIPK2*
PHG-IFG	IFG	GO:0014032	neural crest cell development	32	3	0.00613	*SEMA3D;SEMA7A;PDCD6*

Supplementary Figures

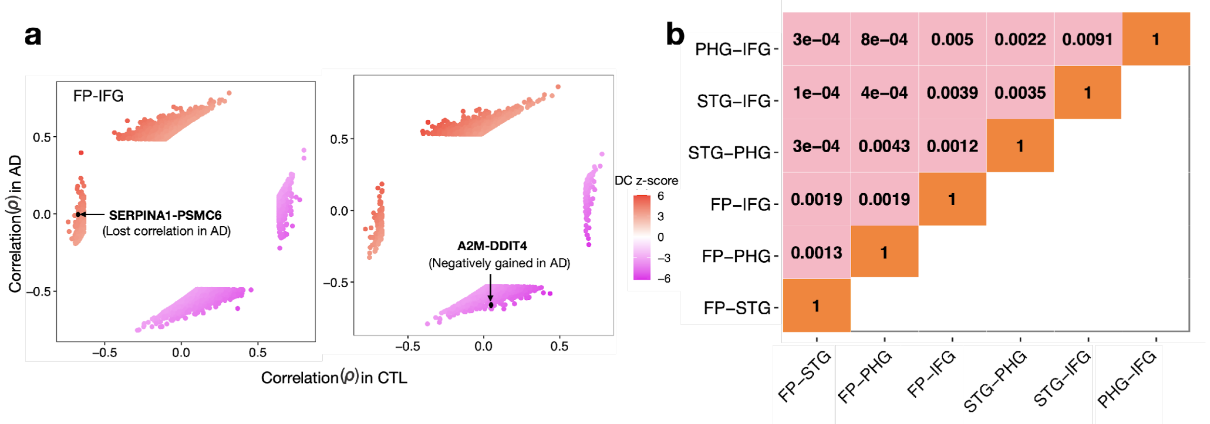

Supplementary Figure 1. Summary of DC analysis across four brain regions.

a. In two example inter-region comparisons, detected DC gene pairs form four distinct clusters in the scatter plot (see also **Suppl. Table 1**), representing different types of change between AD and CTL. Example DC gene pairs pointed in this figure illustrate two of these types/clusters of change, and that in **Fig. 1c of main text** illustrate the other two types.

b. DC gene sets across brain inter-region comparison share almost no similarity as indicated by Jaccard index.

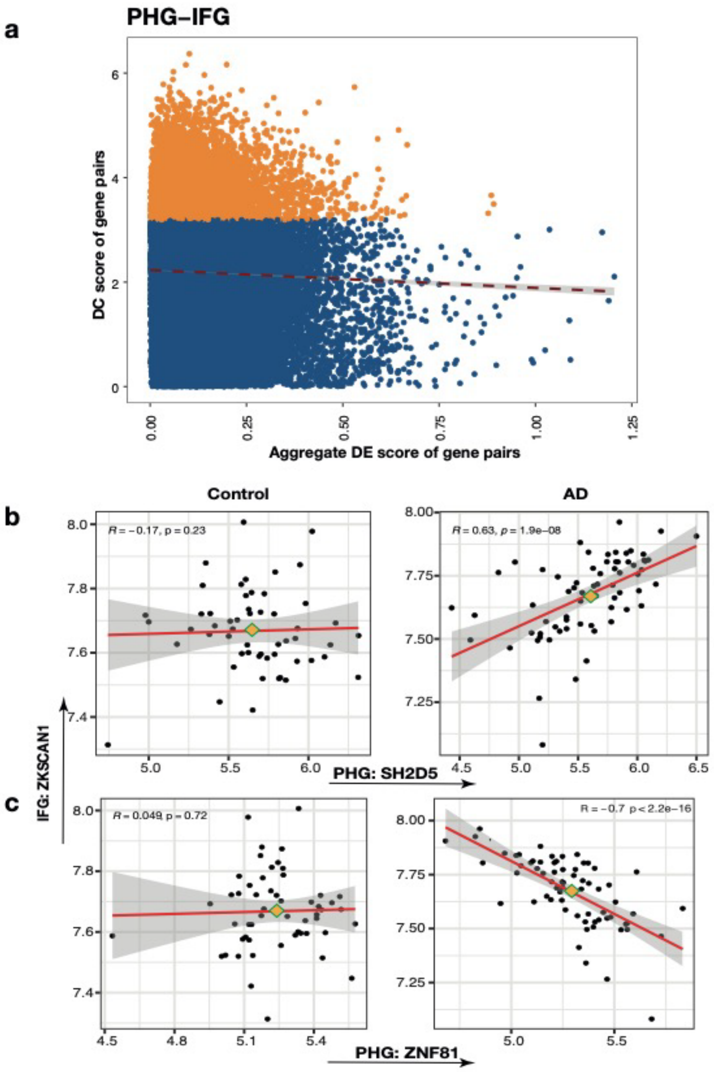

Supplementary Figure 2. DC and DE provide complementary insights.

a. The aggregate DE score (sum of |$\log_2$FC|, DE fold change, of both the genes in a pair) is compared with the DC z-score of the same gene pair. Orange dots signify significant DC, whereas blue dots represent the remaining gene pairs. It is evident that gene pairs with high DC z-scores (orange dots) can rarely achieve high DE aggregate scores, while the DE aggregate score of the remaining gene pairs tested for DC fluctuates.
b. The gene pair SH2D5-ZKSCAN1 in PHG-IFG brain region interaction is correlated in AD and not in control. Their differential correlation is not accompanied by any differential expression (as the yellow diamond indicating the average expression of genes do not appear to change between AD vs. CTL)
c. Similar observation as seen in panel b.

These plots show the linear regression model fit to the data and the 95% confidence interval around this model as a line and grey band respectively (see R ggplot2 geom_smooth function).

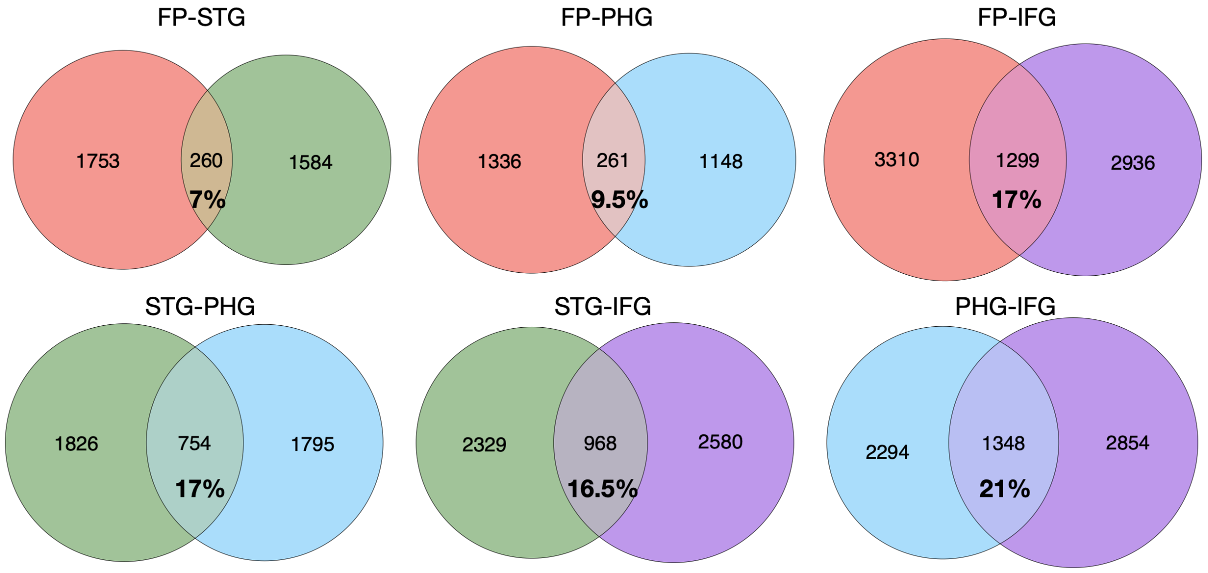

Supplementary Figure 3. Number and percentage of common DC genes across each brain inter-region comparison. Percentage is calculated based on total number of genes present in each inter-region comparison, e.g., in FP-STG DC relation there are 1753 genes exclusive to FP and 1584 genes exclusive to STG and 260 common between FP and STG, so in total there are 3597 genes (see also **Suppl. Table 2**).

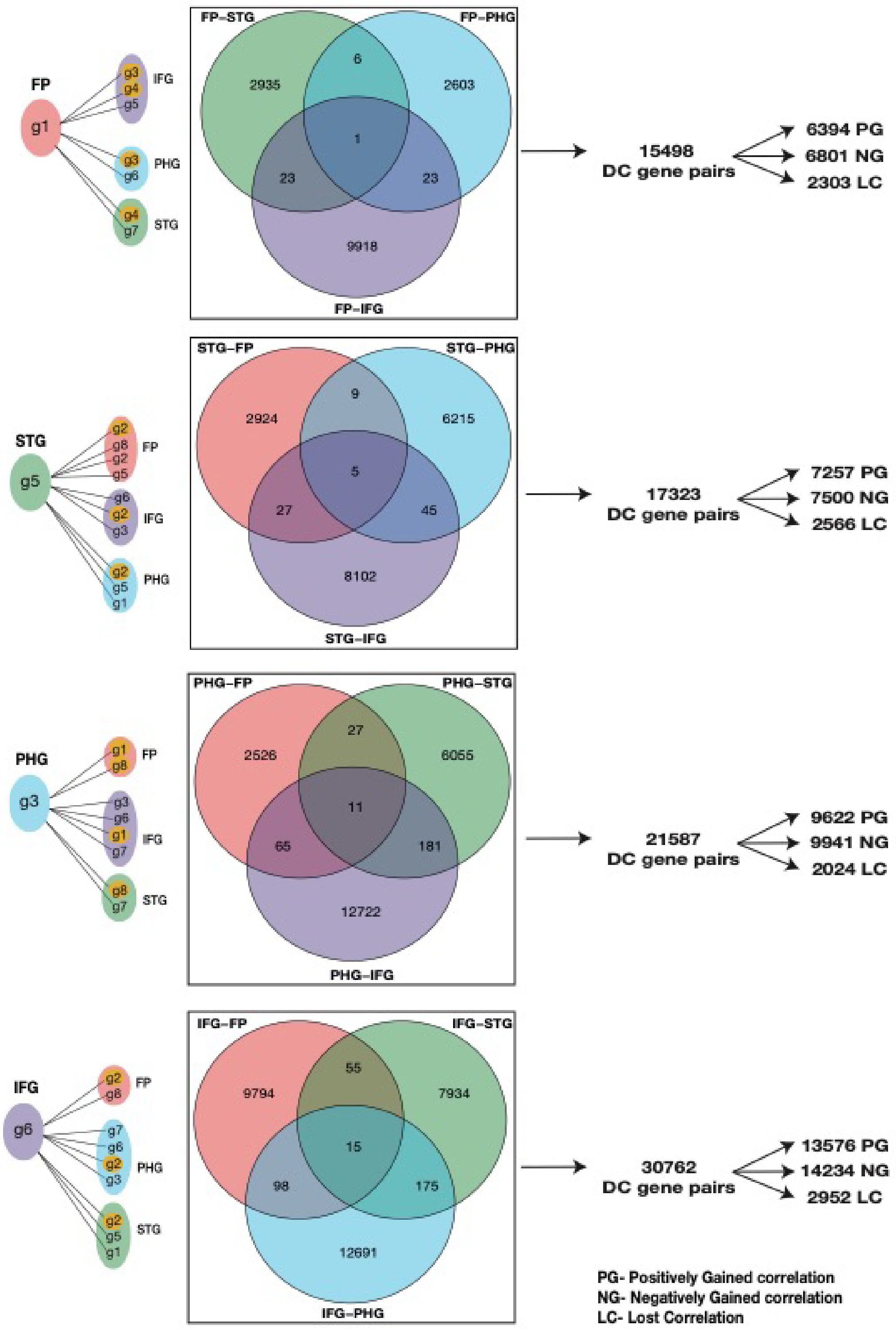

Supplementary Figure 4. Per brain region analysis. This figure emphasizes that only a few DC edges are common between two inter-brain-region comparisons. Intriguingly, all the common DC edges consistently exhibit the same direction. Furthermore, for each brain region, we aggregated all the DC edges and categorized them into positively gained correlation (PG), negatively gained correlation (NG), and lost correlation (LC).

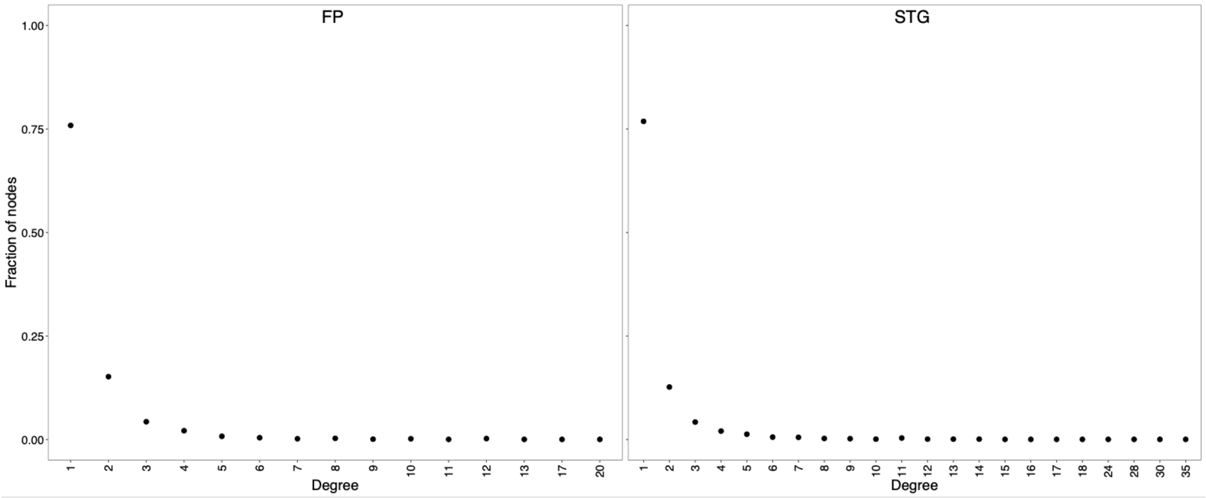
FP
STG
Fraction of nodes
1.00
0.75
0.50
0.25
0.00
1 2 3 4 5 6 7 8 9 10 11 12 13 17 20
1 2 3 4 5 6 7 8 9 10 11 12 13 14 15 16 17 18 24 28 30 35
Degree
Degree

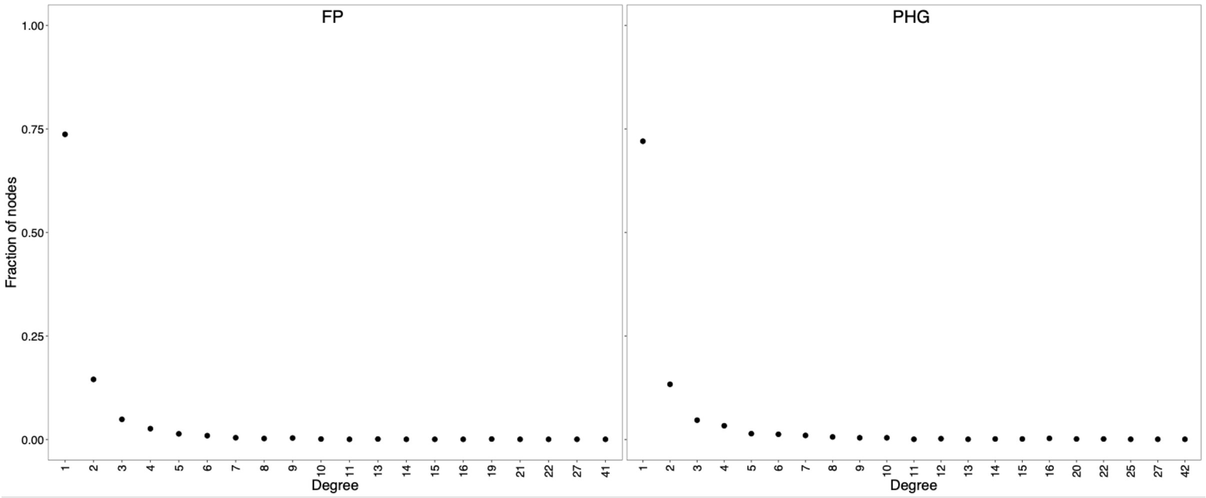
FP
PHG
Fraction of nodes
1.00
0.75
0.50
0.25
0.00
1 2 3 4 5 6 7 8 9 10 11 13 14 15 16 19 21 22 27 41
1 2 3 4 5 6 7 8 9 10 11 12 13 14 15 16 20 22 25 27 42
Degree
Degree

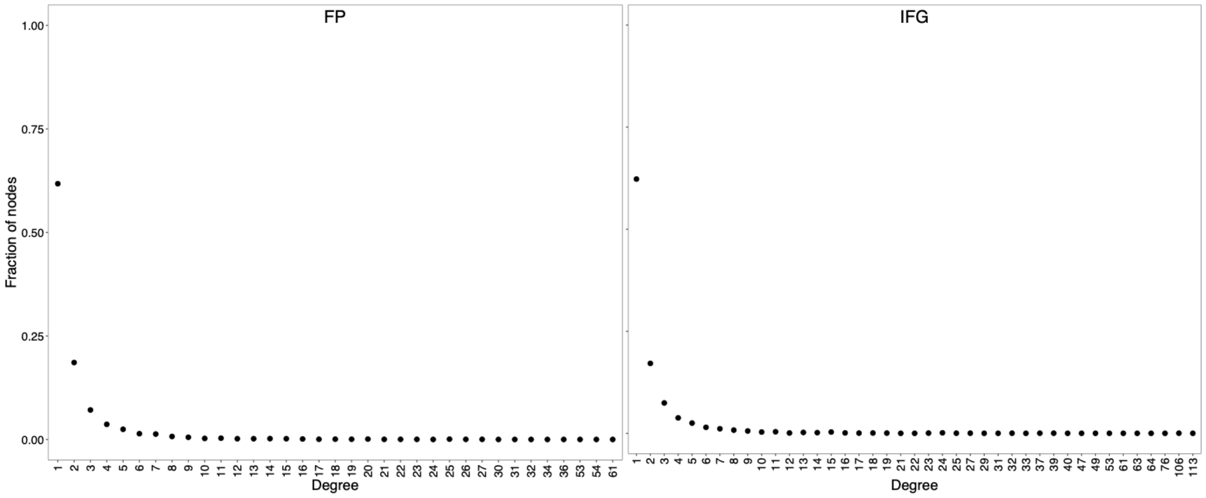
FP
IFG
Fraction of nodes
1.00
0.75
0.50
0.25
0.00
Degree
Degree

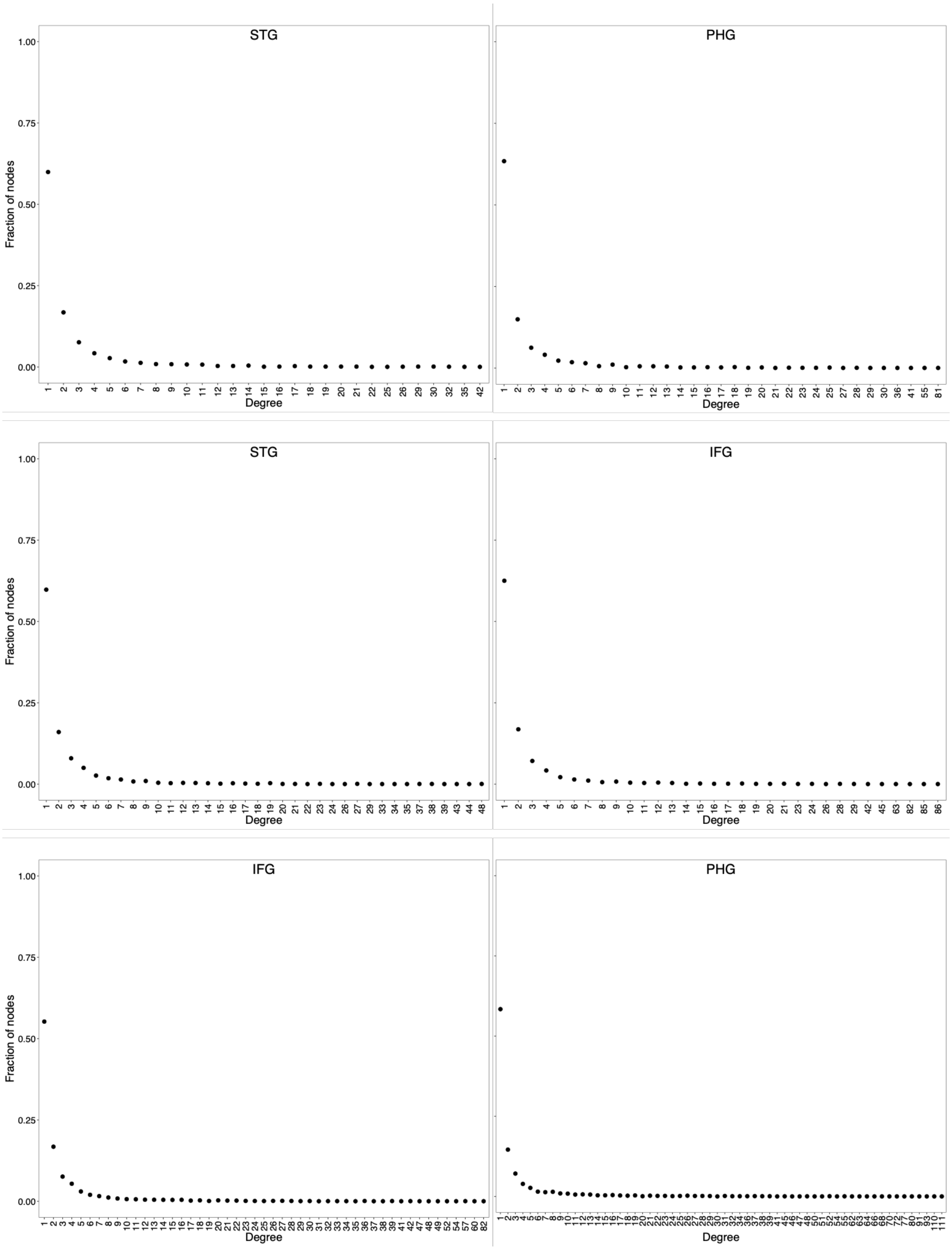

Supplementary Figure 5. Degree distribution of genes in a brain region in each inter-region comparison. A degree of a gene in an inter-region comparison is the number of DC relations/interactions that this gene participates in.

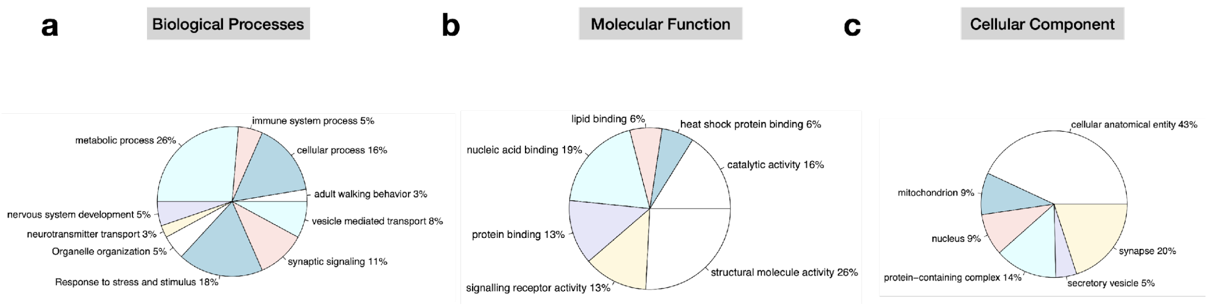

Supplementary Figure 6. Summary of GO categories' terms enriched in our DC modules. Percentage of modules enriched per gene ontology term is depicted.

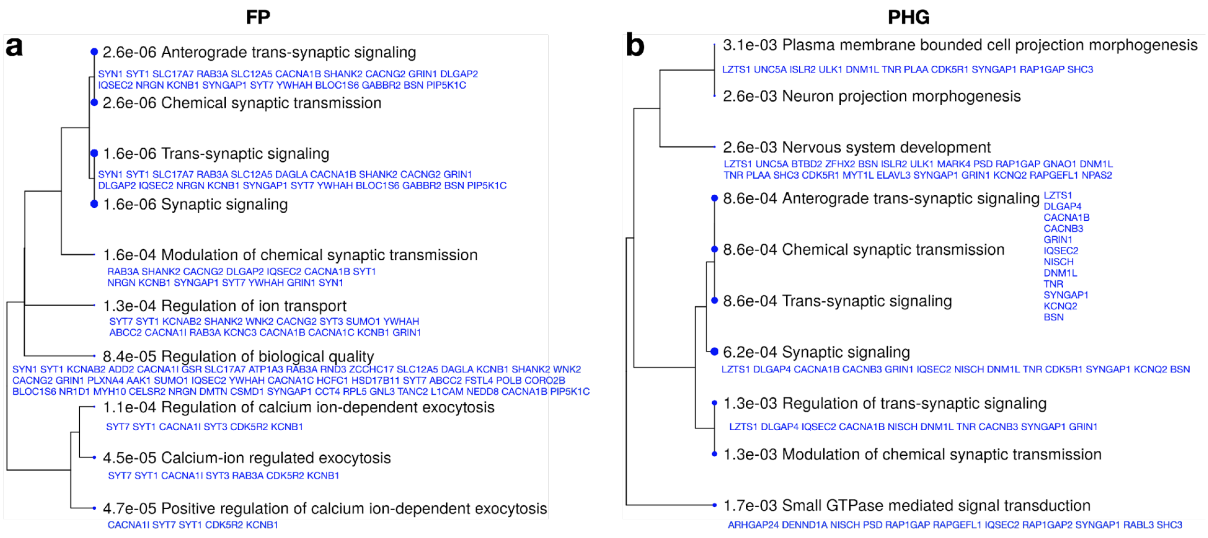

Supplementary Figure 7. Hierarchical clustering tree representing GO_BP (Gene Ontology Biological Process) terms enriched in mod715. (a) GO terms enriched in the FP gene set of our DC module FP-PHG::mod715 are shown, along with the mod715 genes overlapping with the corresponding GO term. Genes common to 2 terms are noted once, either in-between two terms or at the side. The hierarchical clustering tree summarizes the correlation among significant BPs. Size of a solid circle indicates the significance (negative $\log_{10}$ p-value) of the corresponding enrichment. Bigger dots indicate more significant P-values. (b) Similar results for GO terms enriched in the PHG side of mod715.

(Generated using ShinyGO v0.66 online platform)

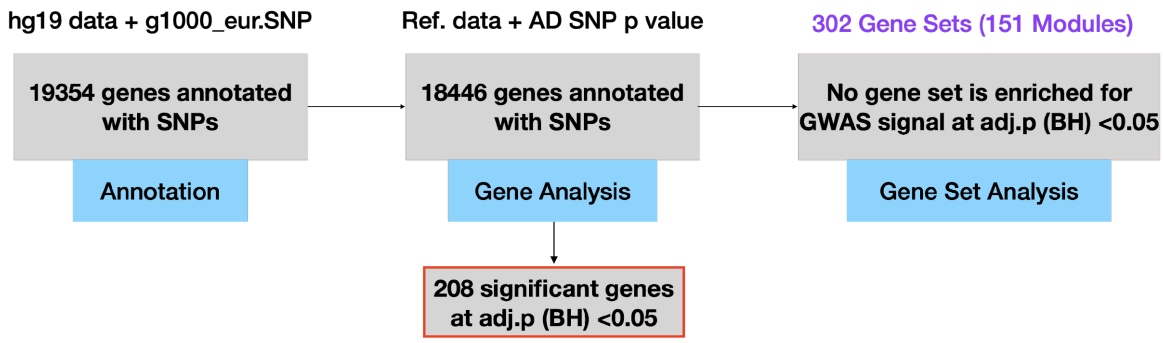

Supplementary Figure 8. Enrichment of DC modules for GWAS signals - Workflow. Schematic of the steps involved in MAGMA gene-level and gene-set-level analysis, that tests whether gene sets are enriched for AD-GWAS (Genome-wide association studies)-signal (see also Methods). (BH method= Benjamini and Hochberg method, p.adj= adjusted P value).

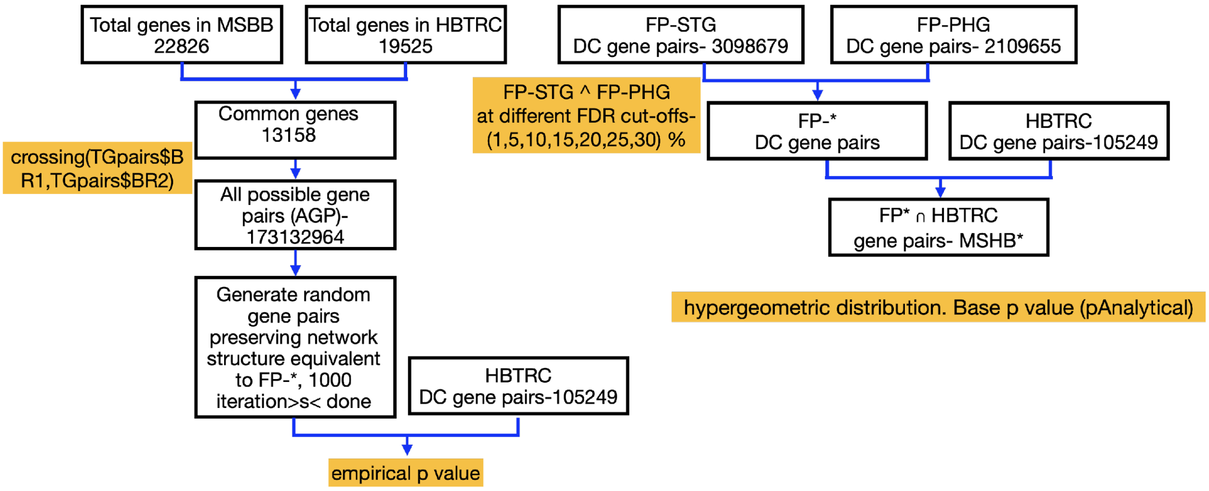

Supplementary Figure 9. Schematic of replication testing of DC results found in our discovery cohort (MSBB) in an independent validation cohort (HBTRC).

DLPFC in HBTRC was closest to FP in MSBB. So, we focused on FP-centred DC pairs and tested only them for replication in the HBTRC cohort. We followed this strategy as there are a very few human cohorts with transcriptomic data on the same brain regions as MSBB at sufficient sample sizes (>= 30). Another challenge we faced during replication is that DC gene pairs in MSBB cohort across brain inter-region comparison share almost no similarity at FDR 1% (**Suppl. Fig. 1b**). So, we decided to relax the cut-off from 1% to different cut-off values ranging from 1% to 30% to obtain sufficient number of common DC pairs between FP-STG and FP-PHG. This resulting set of observed FP-centered DC edges (195,102 edges at FDR 30%) had an overlap of 407 pairs against HBTRC DC pairs (105,249 DC pairs at FDR 1%), and this replication overlap was significantly better than that of random gene pairs with the same size and network structure as the observed DC pairs (p<0.0009).

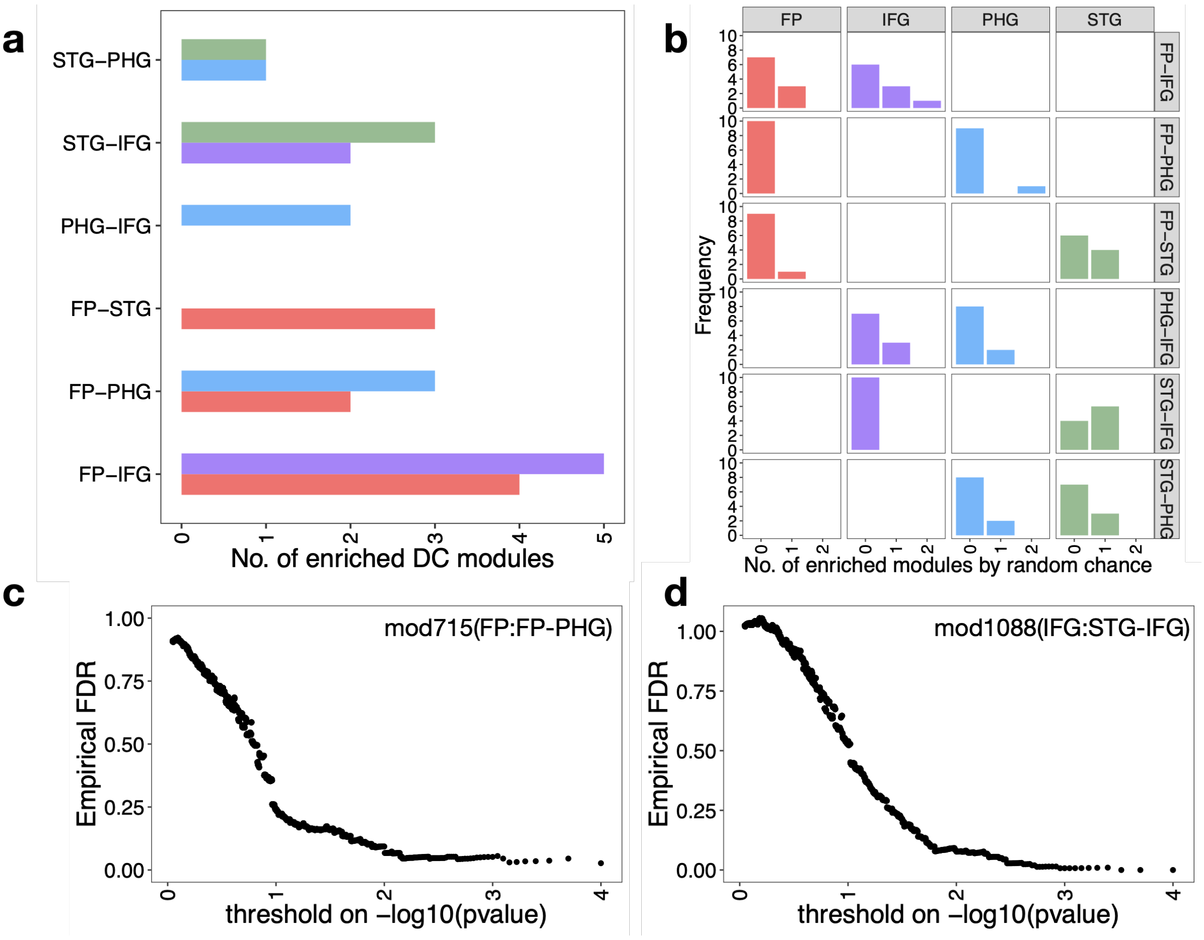

Supplementary Figure 10. Robustness check for DC module enrichment

(a) Number of DC modules enriched for each brain region per inter-region comparison in ORA are represented.

(b) Number of random modules enriched for each brain region per inter-region comparison in ORA are represented. Summary of 10 permutations are represented here.

(c) & (d) Empirical FDR for 2 representative modules are represented in the form of scatter plots.

Supplementary Files

Suppl File 1: Inter-brain-region DC gene pair interactions- All DC gene pairs in six inter-brain-region comparisons at FDR 1% are noted. The first sheet contains the header description. From the second sheet onwards all the 6 inter-brain-region comparisons are included, in 6 separate sheets. The last sheet contains the top 20 DC hub genes from 6 Inter-DC networks.

Suppl File 2: Module details- 302 Genes sets (151 modules) are included in this .gmt file. The first column contains the gene set name [e.g., 1015BR1BM10-22 means module 1015 belongs to brain region 1, BR1 (BM10 or FP in this case) in the inter-brain-region comparison BM10-22 or FP-STG]. The second column in a .gmt file is meant for description. No description is included, hence is denoted with "na". From the third column onwards all the gene entrez ids for the respective gene set are noted.

Suppl File 3: Functional Enrichment- Enriched functional profiles of DC modules. Gene Ontology Molecular Functions & Cellular Components and KEGG & Reactome pathways are represented in the functional profile of each module. The first sheet contains the legend, and the second sheet has a summary of the number of modules enriched for each category & the parameter description. From the third sheet onwards results for all the 6 inter-brain-region comparisons are included, in 6 separate sheets.

Suppl File 4: GO_BP Enrichment of GOC/LOC DC edges- Enriched Gene Ontology Biological Processes (GO_BP) for PG, NG and LC DC edges are noted.

Suppl File 5: GO_BP Enrichment- Enriched Gene Ontology Biological Processes (GO_BP) are represented. The first sheet contains the legend and the parameter description. The second sheet contains all the module enrichment results.

Supp. File 6: Genes involved in Mitochondrial cascade hypothesis- This file summarizes the DC interactions in each brain region pair involving different Sirtuins [SIRTs] genes, which helps in mitophagy regulation.

Suppl File 7: Customized gene sets- This .gmt file contains the 10 signaling based functions along with the genes associated for each term. The first column contains the gene set name [e.g., SNP, neurotransmission, cell-cell signaling etc.]. The second column in a .gmt file is meant for description. No description is included, hence is denoted with "na". From third column onwards all the gene symbols for the respective gene set are noted.

All the supplementary files are available at the following link: https://drive.google.com/drive/folders/1HqVG48aC6rdPT1RJIQB8tW9PcAblKitt?usp=sharing .
